# Long non-coding RNA KCNQ1OT1 alleviates postmenopausal osteoporosis by modulating miR-421-3p/mTOR axis

**DOI:** 10.1038/s41598-023-29546-4

**Published:** 2023-02-09

**Authors:** Ziyu Wang, Hengshuo Zhang, Qinghui Li, Lu Zhang, Lu Chen, Hongliang Wang, Yunzhen Chen

**Affiliations:** 1 Department of Orthopedics, Qilu Hospital of Shandong University, Jinan, 250012 People’s Republic of China; 2 Cheeloo College of Medicine, Shandong University, Jinan, 250012 People’s Republic of China

**Keywords:** Mechanisms of disease, Epigenetics, Osteoporosis

## Abstract

The prevention and treatment of postmenopausal osteoporosis (PMOP) is a significant public health issue, and non-coding RNAs are of vital importance in this process. In this study, we find that the long non-coding RNA potassium voltage-gated channel subfamily Q member 1 overlapping transcript 1 (lncRNA KCNQ1OT1) can alleviate the ovariectomy-induced (OVX) PMOP in vivo. We determined that over-expression of KCNQ1OT1 could enhance functions of MC3T3-E1 cells, whereas an opposite trend was observed when KCNQ1OT1 was knocked down. Subsequently, miR-421-3p targeting KCNQ1OT1 was detected through a database search, and RNA fluorescent in situ hybridization, RNA immunoprecipitation, dual luciferase reporter assays all verified this relationship. Notably, KCNQ1OT1 stifled the miR-421-3p expression. The inhibition of proliferation, migration, and osteogenic differentiation caused by KCNQ1OT1 knock-down were reversed by an miR-421-3p inhibitor, further confirming the above findings. We verified that miR-421-3p specifically targeted the mammalian target of rapamycin (mTOR), and miR-421-3p inhibitor could reverse the negative effects of small interfering RNA of mTOR (si-mTOR) on MC3T3-E1 cells. Finally, osteoblasts isolated and cultured from OVX mice model and control mice also confirmed the observed trend. In combination, results mentioned above reveal that KCNQ1OT1 regulates MC3T3-E1 cell functions by regulating the miR-421-3p/mTOR axis.

## Introduction

With the aging of global population becoming increasingly heavier, the prevalence and social burden of OP are becoming aggravated, making it a major public health problem^1^. And the majority of OP patients are elderly^2^. In elderly OP patients, OP is four times more common in female than in male, and female patients mainly suffer from postmenopausal osteoporosis (PMOP)^3–5^. Despite there are many options for the treatment of PMOP, concerns about side-effects and lack of sufficient evidence in support of their long-term efficacy lead to poor medication compliance^6–9^. Therefore, more in-depth studies of PMOP at molecular level are required to develop more effective therapeutic methods for PMOP^10,11^.

Long noncoding RNAs (lncRNAs) are characterized as non-protein coding RNAs with transcripts > 200 nucleotides, and lncRNAs are located in nucleus or cytoplasm^12^. LncRNAs localized in the cytoplasm can affect mRNAs at the post-transcriptional level via sponging microRNAs (miRNAs), thereby exert the function of regulation of epigenetic mechanisms^13,14^. Concretely, lncRNAs can bind to particular miRNA reaction elements to prevents miRNAs from binding to their target mRNAs^15^. Previous studies proved that a lot of lncRNAs, as competing endogenous RNAs (ceRNAs), are associated with proliferation, apoptosis and differentiation of osteoblast and osteoclast by interacting with miRNAs^16–18^. Therefore, abnormalities of lncRNAs are closely related to the occurrence of OP via lncRNA/miRNA/mRNA axis^19–21^.

LncRNA potassium voltage-gated channel subfamily Q member 1 overlapping transcript 1 (KCNQ1OT1) is placed in the chromosomal region 11p15.5^22^. Previous studies proved that KCNQ1OT1 promoted differentiation of osteoblast in mouse mesenchymal stem cells (mMSCs) and human bone marrow mesenchymal stem cells (hBMSCs)^23,24^. No relevant studies have been conducted to investigate the combined effects of KCNQ1OT1 on the cell viability and differentiation capacity of MC3T3-E1 cells. Moreover, the expression changes of KCNQ1OT1 in PMOP patients and its effect on PMOP mice model are not investigated. Therefore, thorough study of the comprehensive influences of KCNQ1OT1 on osteoblast proliferation, migration, differentiation and how KCNQ1OT1 effects PMOP mice model is required.

MicroRNAs (miRNAs) are a category of non-coding RNAs, generally with the length of 18–25 nucleotides^25^. Up to 60% of human protein-encoding messenger RNAs (mRNAs) are considered to be regulated by miRNAs^26^. Many research showed that miRNAs can regulate osteogenic and osteoclast differentiation, which in turn affects bone remodeling^27,28^. As for miRNA-421-3p (miR-421-3p), there is no study on how miR-421-3p is involved in the regulation the functions of osteoblasts and osteoclasts.

The mammalian target of rapamycin (mTOR) could senses extracellular energy states and regulate cell proliferation and metabolism in various cells and tissues^29^. Previous studies showed that mTOR can promote osteogenic differentiation^30–32^.

In this study, we used bioinformatics database and determined that miR-421-3p had binding sites with mTOR. Similarly, binding sites also exist in KCNQ1OT1 and miR-421-3p. However, the interactions among KCNQ1OT1, mTOR and miR-421-3p in PMOP has not been investigated. Our study highlights the effects of KCNQ1OT1/miR-421-3p/mTOR axis in osteogenic differentiation for the first time and can provide new ideas and methods for the treatment of PMOP.

## Results

### KCNQ1OT1 enhances the proliferation, migration and osteogenic differentiation of MC3T3-E1 cells

**Figure 1:**
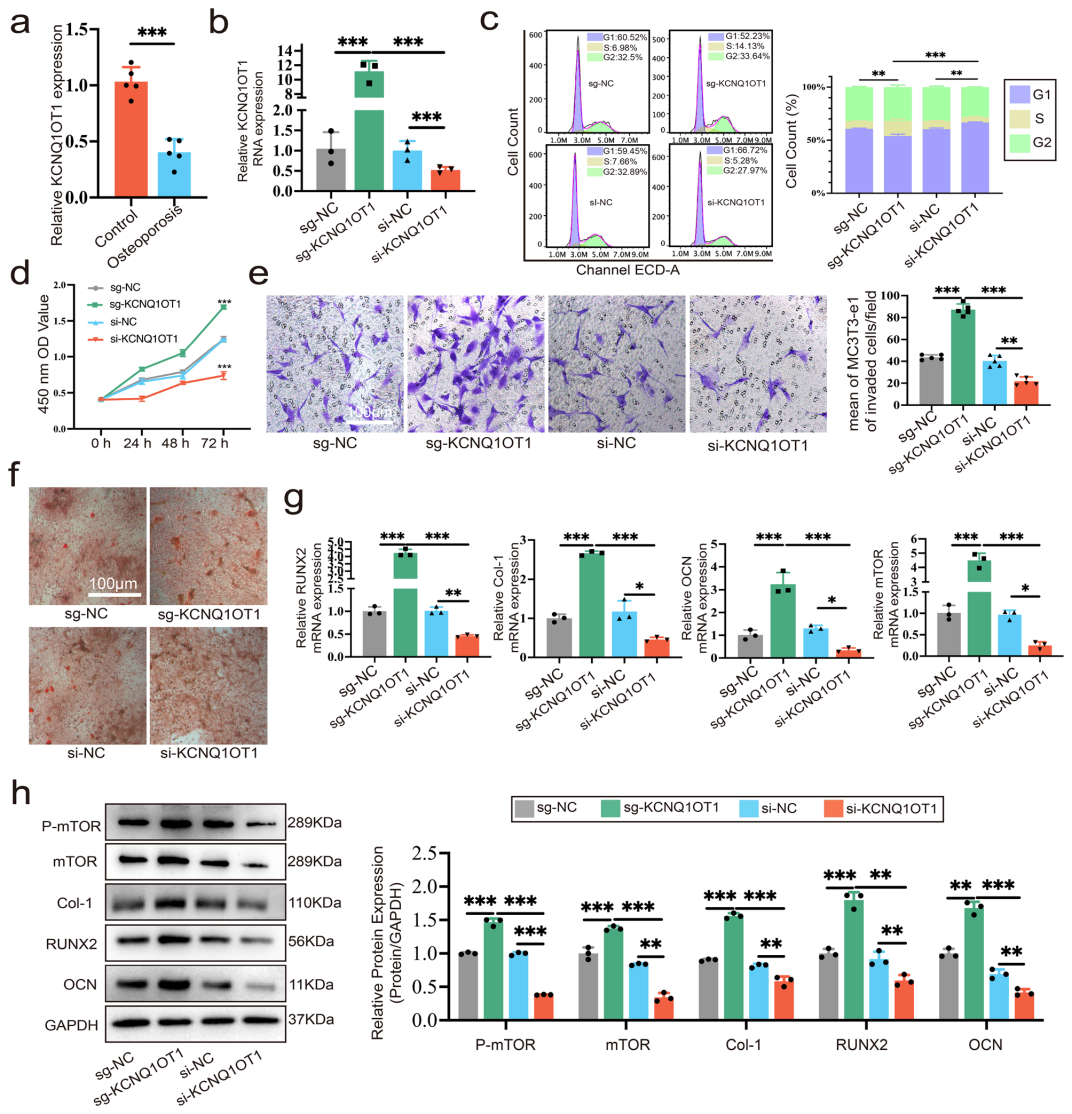
KCNQ1OT1 enhances the proliferation, migration and osteogenic differentiation of MC3T3-E1 cells. MC3T3-E1 cells were transfected with sg-KCNQ1OT1, sg-NC, si-KCNQ1OT1, si-NC, respectively. (**a**) Relative KCNQ1OT1 expression in bone tissues of patients with osteoporosis and controls without osteoporosis by qRT-PCR (N = 5). (**b**) RNA expression of KCNQ1OT1 were examined by qRT-PCR analysis to detect the transfection efficiency. (**c**) MC3T3-E1 cell cycle analysis after PI staining. (**d**) Through the CCK-8 assay, the impact of KCNQ1OT1 on the ability of MC3T3-E1 cells to proliferate was evaluated. (**e**) MC3T3-E1 cells migration ability was tested using a transwell migration experiment to show the impacts of KCNQ1OT1. (**f**) Representative images of MC3T3-E1 cells stained with alizarin red after transfection and 14 days of osteogenic induction. (**g**) RNA expression of RUNX2, Col-1, OCN and mTOR were examined by qRT-PCR analysis. (**h**) Protein expression levels of pmTOR, mTOR, Col-1, RUNX2 and OCN were analyzed by Western blot after transfection and osteogenic differentiation for seven days. Uncropped Western blot images are shown in Supplementary Fig. 5. *P < 0.05, **P < 0.01, ***P < 0.001.

Patients with PMOP showed significantly decreased levels of lncRNA KCNQ1OT1 expression in their bone tissue, compared to the control group (Fig. 1a). Therefore, in order to further explore the effect of KCNQ1OT1 on the biological function of MC3T3-E1 cells in vitro, small interfering RNA (siRNA) targeting KCNQ1OT1 (si-KCNQ1OT1), over-expression vector virus of KCNQ1OT1 (sg-KCNQ1OT1) and their negative controls (NCs) were transfected into MC3T3-E1 cells respectively (Fig. 1b). Cell cycle analysis showed that si-KCNQ1OT1 arrested cells in the G1 phase of mitosis compared with si-NC, while sg-KCNQ1OT1 promoted cell entry into S and G2 phases, which was facilitated for cell proliferation (Fig. 1c). KCNQ1OT1 over-expression significantly increased the MC3T3-E1 cells growth in comparison to NC, according to Cell Counting Kit-8 (CCK-8) assay (Fig. 1d). Transwell migration assay showed that sg-KCNQ1OT1 obviously enhanced the MC3T3-E1 cells’ migration capacity, while si-KCNQ1OT1 was not conducive to cell migration (Fig. 1e). In order to verify how KCNQ1OT1 affected osteogenic differentiation, MC3T3-E1 cells were cultured with osteogenic induction medium after transfection. Alizarin red staining revealed that KCNQ1OT1 overexpression increased mineralization after 14 days of induction (Fig. 1f). Then, the expression of collagen-1 (Col-1), runt-related transcription factor 2 (RUNX2) and osteocalcin (OCN) were then investigated during osteogenic differentiation using qRT-PCR, Western blot, and immunofluorescence (IF) staining. In detail, the mRNA expression levels of the osteogenic genes Col-1, RUNX2 and OCN were increased in sg-KCNQ1OT1 group (Fig. 1g), and sg-KCNQ1OT1 upregulated the protein expression of osteogenesis-related genes Col-1, RUNX2 and OCN (Fig. 1h).The fluorescence intensity of mTOR and RUNX2 of sg-KCNQ1OT1 group was the highest, indicating that KCNQ1OT1 promoted osteogenic differentiation of MC3T3-E1 cells (Supplementary Fig. 1a, b). These results prove that KCNQ1OT1 enhances the proliferation, migration, and osteogenic differentiation of MC3T3-E1 cells. In addition, we proved that interference or overexpression of KCNQ1OT1 was able to inhibit or enhance the expression of mTOR and phospho-mTOR (pmTOR). In order to determine how it adjusted mTOR and its phosphorylation, the following experiments were needed to prove.

### KCNQ1OT1 directly interacts with miR-421-3p

**Figure 2:**
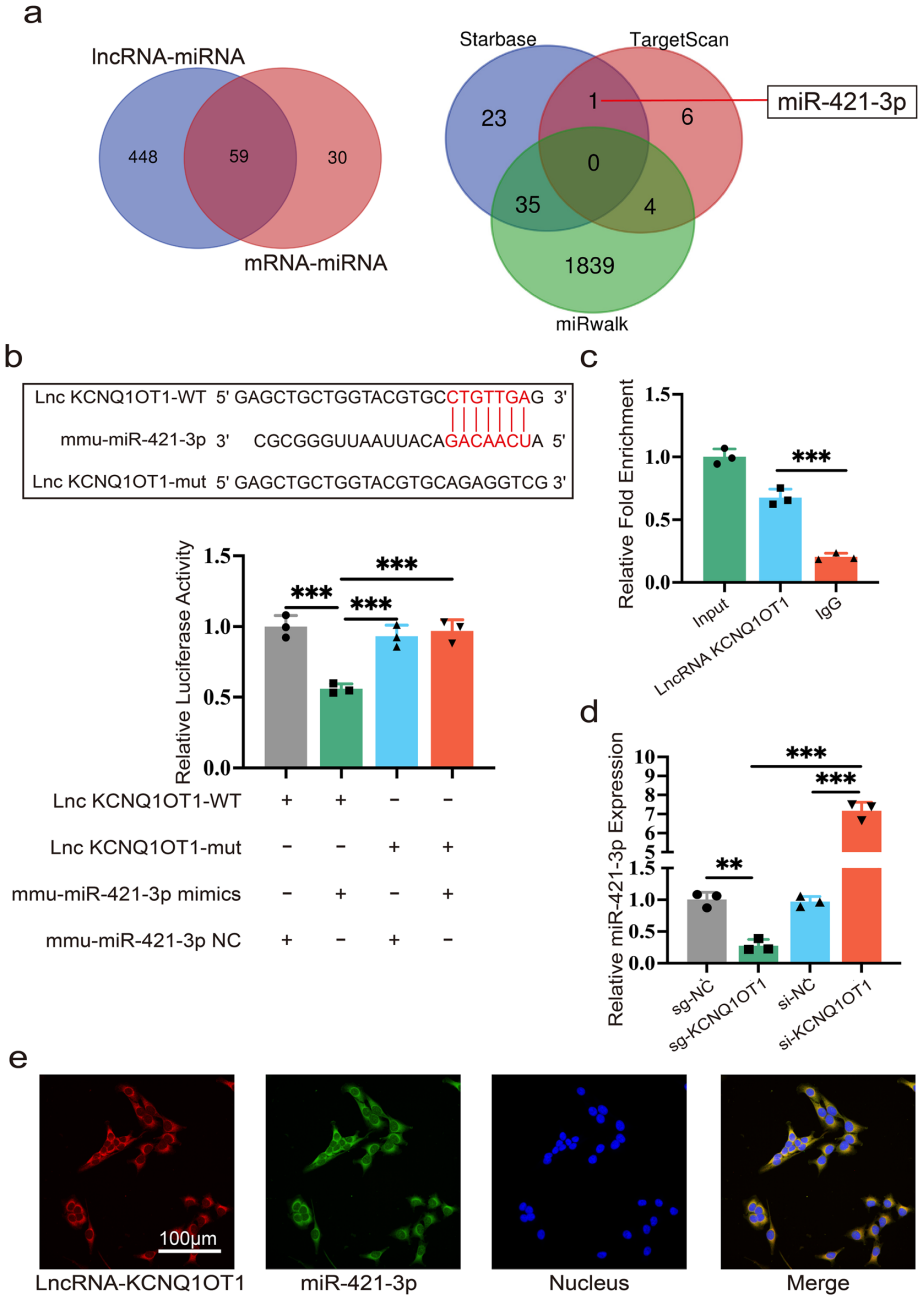
KCNQ1OT1 directly interacts with miR-421-3p. (**a**) Bioinformatic databases predicted that miR-421-3p could be a possible target of KCNQ1OT1. (**b**) The binding site between KCNQ1OT1 and miR-421-3p was predicted by Starbase. After HEK-293T cells transfected with mmu-421-3p NC or mmu-miR-421-3p mimics in wild-type (WT) and mutanttype (mut) of KCNQ1OT1, a dual luciferase reporter assay was performed. (**c**) The RIP assay was completed using input from cell lysate, IgG, or anti-Ago 2, relative RNA expression levels were presented as fold enrichment in Ago2 relative to IgG IP. (**d**) Relative miR-421-3p expression in MC3T3-E1 cells transfected with sg-KCNQ1OT1, sg-NC, si-KCNQ1OT1 and si-NC. (**e**) The location of KCNQ1OT1 and miR-421-3p were both in cytoplasm of MC3T3-E1 cells via RNA-FISH assay. **P < 0.01, ***P < 0.001.

Cytoplasmic lncRNAs have the ability to adsorb miRNAs like sponges, reducing endogenous miRNA binding to target genes at the post-transcriptional level. We used the online bioinformatic database Starbase to predict miRNAs that interacted with KCNQ1OT1, as well as those that might regulate mTOR. After taking the intersection of the results, we found that 59 miRNAs were associated with both KCNQ1OT1 and mTOR (Fig. 2a). Then, TargetScan and miRwalk were used to predict miRNAs that regulated mTOR, from which 10 and 1878 miRNAs were found, respectively. These miRNAs were used to generate a Venn diagram, together with the 59 miRNAs predicted by Starbase, in which the intersection of Starbase and miRwalk included 35 miRNAs, and the intersection of Starbase and TargetScan included 1 miRNA, which was miR-421-3p (Fig. 2a). Interestingly, a highly conserved binding region between KCNQ1OT1 and miR-421-3p was discovered. According to the dual luciferase assay, the miR-421-3p mimic significantly reduced the luciferase activity of HEK-293 T cells in the KCNQ1OT1- wild type (WT) group, whereas no discernible changes in luciferase activity were found in the KCNQ1OT1-mutant type (mut) group (Fig. 2b). Furthermore, RNA immunoprecipitation (RIP) assay showed that, compared to IgG, KCNQ1OT1 enhanced the enrichment of the miR-421-3p ribonucleoprotein complex including Ago2 (Fig. 2c). The qRT-PCR results demonstrated that si-KCNQ1OT1 enhanced the expression of miR-421-3p, while sg-KCNQ1OT1 inhibited it (Fig. 2d). To further demonstrate the location at which KCNQ1OT1 and miR-421-3p interacted with each other in the cell, RNA fluorescent in situ hybridization (RNA-FISH) was performed. The results revealed that both KCNQ1OT1 and miR-421-3p were mainly present in the cytoplasm (Fig. 2e). All of the aforementioned evidence demonstrates that KCNQ1OT1 acted as a miR-421-3p sponge.

### miR-421-3p suppresses the viability of MC3T3-E1 cells

**Figure 3:**
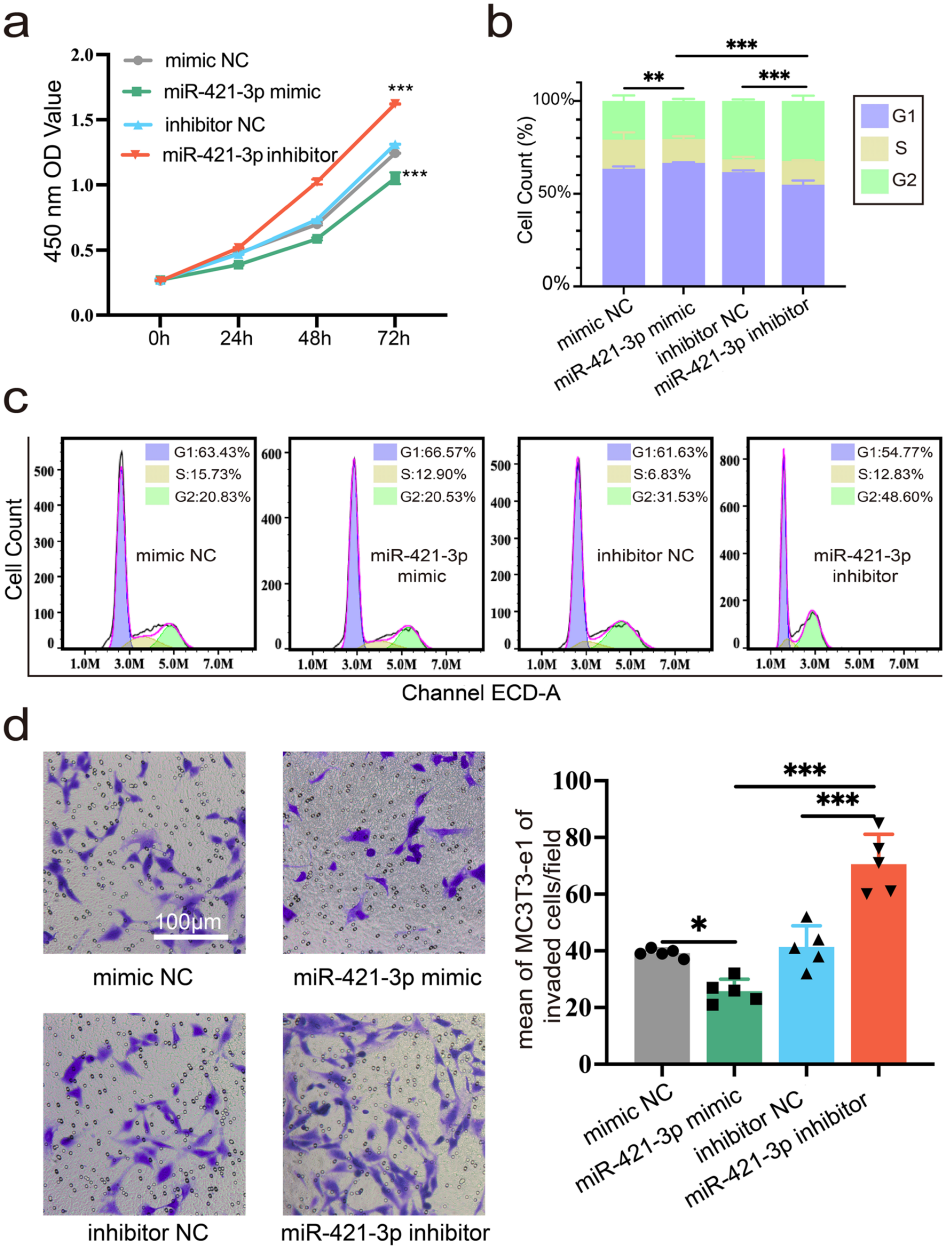
miR-421-3p suppresses the viability of MC3T3-E1 cells. MC3T3-E1 cells were transfected with miR-421-3p mimic, mimic NC, miR-421-3p inhibitor or inhibitor NC for 72 h, respectively. (**a**) CCK-8 assay was used to determine how the mimic and inhibitor miR-421-3p affected the ability of cells to proliferate. (**b**, **c**) Cell cycle analysis in MC3T3-E1 cells stained with PI. (**d**) Transwell migration assay showed the effects of the mimic and inhibitor of miR-421-3p on migration ability of MC3T3-E1 cells. *P < 0.05, **P < 0.01, ***P < 0.001.

CCK-8 assay showed that the inhibitor of miR-421-3p greatly enhanced the proliferation of MC3T3-E1 cells, while the mimic of miR-421-3p was unfavorable for cell proliferation (Fig. 3a). A cell cycle assay was used to explore the reasons for the above phenomenon. The results revealed that miR-421-3p mimic caused cells to be arrested in the G1 phase of mitosis, compared to mimic NC, and affected chromosome replication, thereby inhibiting cell mitosis and proliferation. On the contrary, the inhibitor of miR-421-3p significantly increased cells in G2 phase, thus promoting cell mitosis and proliferation (Fig. 3b,c). Transwell assay showed that the mimic of miR-421-3p suppressed cell migration compared to NC and inhibitor (Fig. 3d). The results mentioned above reveal that miR-421-3p can suppress the proliferation and migration ability of MC3T3-E1 cells.

### miR-421-3p suppresses osteogenic differentiation of MC3T3-E1 cells

**Figure 4:**
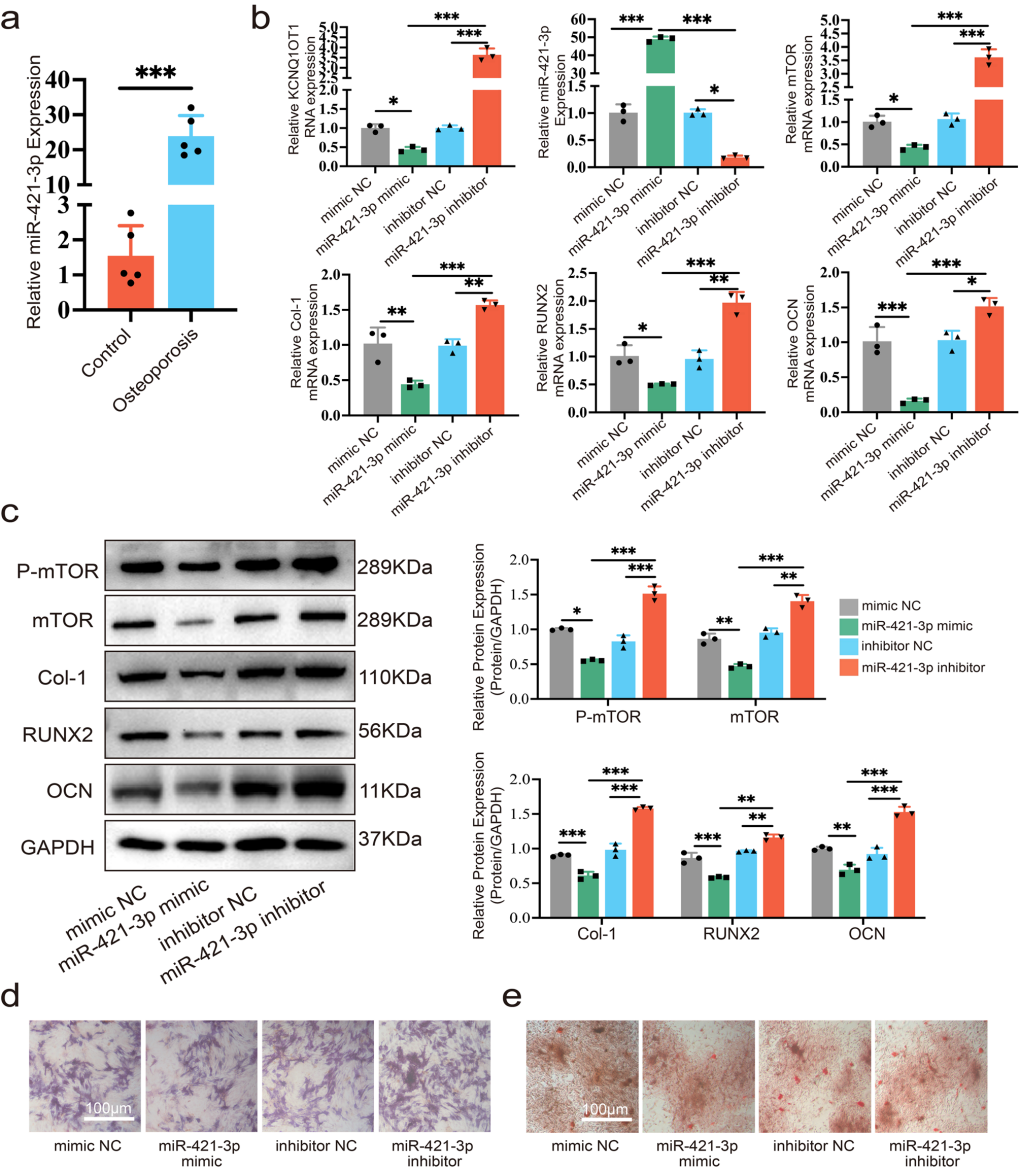
miR-421-3p suppresses MC3T3-E1 cells’ osteogenic differentiation. MC3T3-E1 cells were transfected with miR-421-3p mimic, mimic NC, miR-421-3p inhibitor or inhibitor NC, respectively. (**a**) Relative expression of miR-421-3p in bone tissue of PMOP patients and controls without osteoporosis was performed by qRT-PCR (N = 5). (**b**) RNA expression of Col-1, OCN, RUNX2, KCNQ1OT1, miR-421-3p and mTOR was tested by qRT-PCR. (**c**) Protein expression levels of pmTOR, mTOR, Col-1, RUNX2 and OCN were analyzed by Western blot after transfection and osteogenic differentiation for seven days. Uncropped Western blot images are shown in Supplementary Fig. 6. (**d**) ALP staining showed ALP levels of MC3T3-E1 cells after transfection and osteogenic differentiation for seven days. (**e**) Alizarin Red staining of MC3T3-E1 cells after transfection and osteogenic differentiation for 14 days. *P < 0.05, **P < 0.01, ***P < 0.001.

In terms of subjects, the expression of miR-421 in osteoporosis patients was significantly upregulated compared to healthy controls (Fig. 4a). To further investigate how MC3T3-E1 cells' osteogenic differentiation was impacted by miR-421-3p, mimic, inhibitor, and their NCs of miR-421-3p were transfected into MC3T3-E1 cells, respectively. qRT-PCR was used to determine the consequent transfection, and the miR-421-3p expression level was significantly elevated in the mimic-transferred group, whereas it was down-regulated in inhibitor-transferred group (Fig. 4b). qRT-PCR and Western blot revealed that the miR-421-3p mimic decreased the expression of osteogenesis-related markers, such as Col-1, RUNX2, and OCN, while the results of the inhibitor group showed the opposite (Fig. 4b,c). Moreover, we found that changes in the expression level of miR-421-3p affected the expression and phosphorylation of mTOR: the miR-421-3p mimic down-regulated the expression of mTOR and pmTOR, while the inhibitor led to the opposite trend (Fig. 4c). IF staining results of RUNX2 and mTOR were also consistent with the above results (Supplementary Fig. 2a, b). Alkaline phosphatase (ALP) staining showed that ALP levels were raised in the miR-421-3p inhibitor group, compared to NC, and were greatly decreased in the inhibitor group (Fig. 4d). Alizarin Red staining confirmed the ALP staining findings, demonstrating that the miR-421-3p inhibitor group developed the majority of mineralized nodules, while the miR-421-3p mimic group rarely produced any (Fig. 4e). The results presented above reveal that the miR-421-3p suppresses MC3T3-E1 cells’ osteogenic differentiation.

### miR-421-3p regulates the cell viability and osteogenic differentiation of MC3T3-E1 cells by targeting mTOR

**Figure 5:**
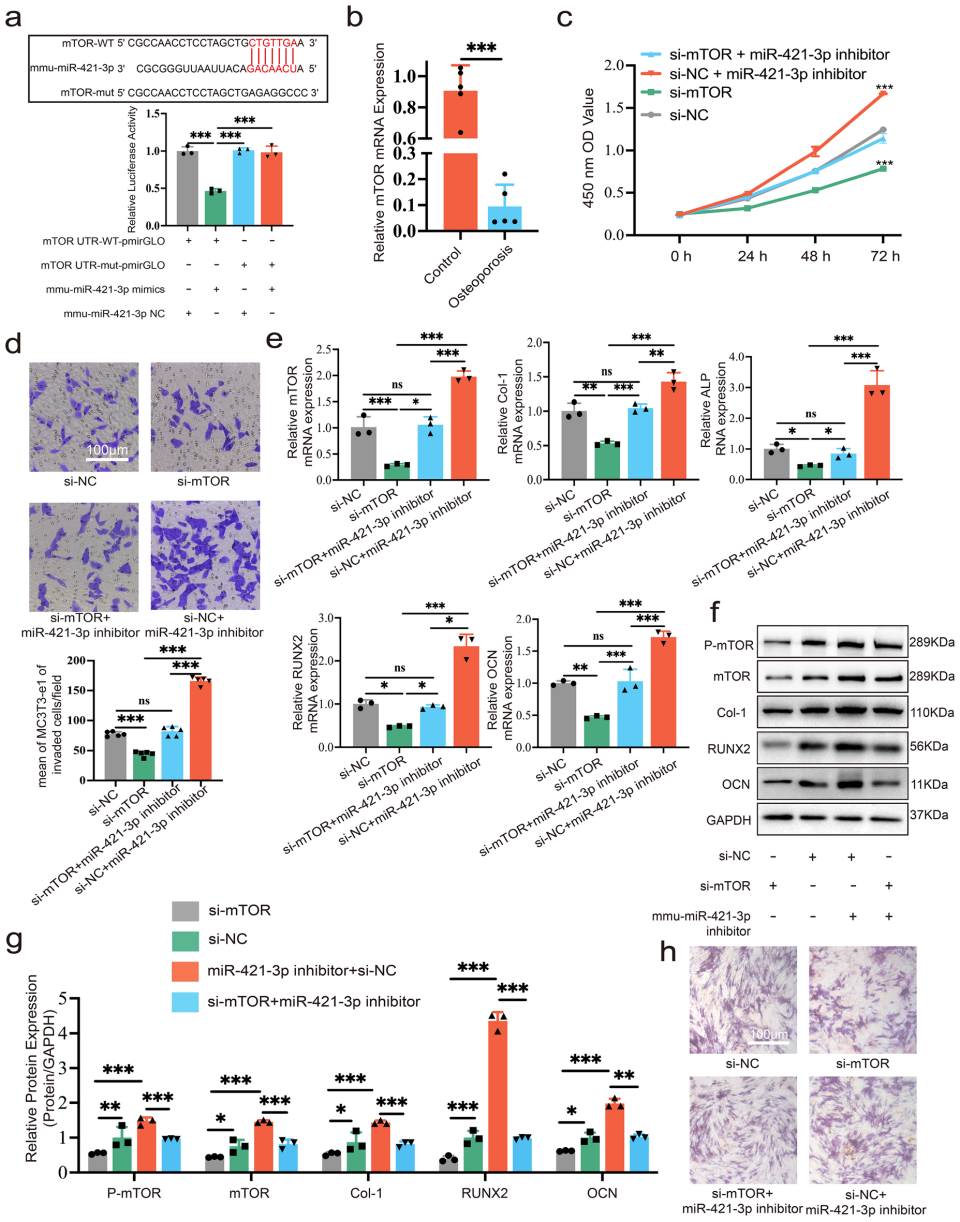
miR-421-3p regulates the cell viability and osteogenic differentiation of MC3T3-E1 cells by targeting mTOR. MC3T3-E1 cells were transfected with si-mTOR, si-NC, si-mTOR + miR-421-3p inhibitor or si-NC + miR-421-3p inhibitor respectively. (**a**) Starbase predicted the location of the miR-421-3p and mTOR binding site. When miR-421-3p mimics or miR-421-3p NC were transfected into mTOR WT and mut of HEK 293T cells, the relative luciferase activity was determined. (**b**) Relative mRNA expression of mTOR in bone tissue of patients with osteoporosis and controls without osteoporosis (N = 5). (**c**) CCK-8 assay showed the changes on proliferation after MC3T3-E1 cells transfection. (**d**) Transwell assay was used to demonstrate the influence on MC3T3-E1 cells' ability to migrate. (**e**) mRNA expression of mTOR, Col-1, RUNX2, ALP and OCN detected by qRT-PCR. (**f**, **g**) Protein expression levels of pmTOR, mTOR, Col-1, RUNX2 and OCN of MC3T3-E1 cells after transfection and cultured in osteogenic induction medium for seven days. Uncropped Western blot images are shown in Supplementary Fig. 7. (**h**) ALP levels of MC3T3-E1 cells after transfection and osteogenic differentiation for seven days was displayed using ALP staining. *P < 0.05, **P < 0.01, ***P < 0.001.

Dual luciferase assay showed that there were no significant changes in the mTOR UTR-mut group, but upregulating miR-421-3p significantly decreased luciferase activity in the mTOR UTR-WT group (Fig. 5a). The above evidence revealed that miR-421-3p targeted mTOR. Moreover, qRT-PCR proved that the mRNA expression of mTOR in bone tissue of PMOP patients was obviously downregulated compared to the controls (Fig. 5b). CCK-8 assay demonstrated that the proliferation of MC3T3-E1 cells was inhibited after transfection siRNA of mTOR (si-mTOR), compared to NC. However, co-transfection of miR-421-3p inhibitor with si-mTOR reversed this inhibition, and the proliferation in the si-mTOR + miR-421-3p inhibitor group showed no statistical difference to si-NC (Fig. 5c). Similarly, si-mTOR suppressed the migration ability of MC3T3-E1 cells, while the miR-421-3p inhibitor reversed this influence (Fig. 5d). As for the effect on osteogenesis, after transfection with si-mTOR, the mRNA and protein expressions of mTOR were obviously down-regulated, and the expression levels of osteogenesis-related markers (i.e., Col-1, RUNX2, ALP and OCN) were also significantly decreased; however, this trend was reversed after transfection with the inhibitor of miR-421-3p (Fig. 5e–g). IF staining was also consistent with the above results (Supplementary Fig. 3a, b). In addition, ALP staining showed that downregulation of mTOR expression impaired the intensity of ALP activity, while co-transfection of miR-421-3p inhibitor with si-mTOR could rescue it (Fig. 5h). Combining the previous experimental results regarding the expression changes of mTOR and pmTOR with the mimic and inhibitor miR-421-3p transfected, and in view of the evidence mentioned above, we determined that miR-421-3p could regulate the cell viability and osteogenic differentiation of MC3T3-E1 cells by targeting mTOR.

### KCNQ1OT1 enhances cell proliferation, migration, and osteogenic differentiation by up-regulating mTOR via sponging miR-421-3p

**Figure 6:**
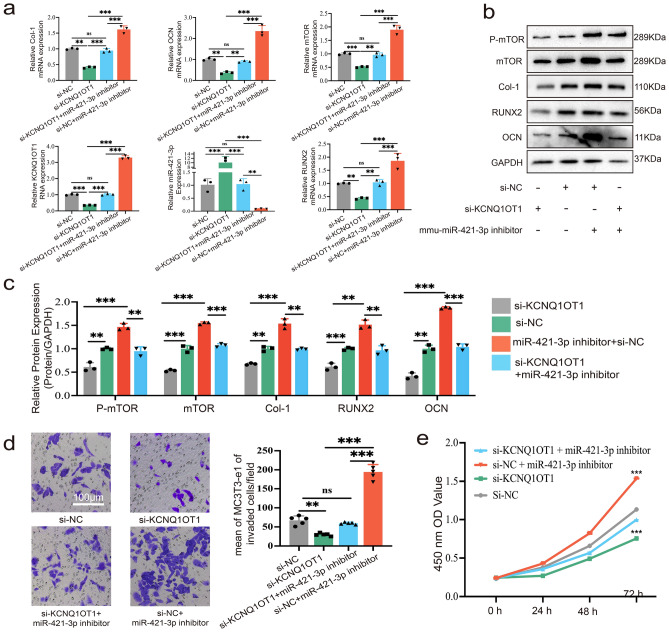
KCNQ1OT1 enhances cell proliferation, migration, and osteogenic differentiation by up-regulating mTOR via sponging miR-421-3p. MC3T3-E1 cells were transfected with si-KCNQ1OT1, si-NC, si-KCNQ1OT1 + miR-421-3p inhibitor or si-NC + miR-421-3p inhibitor respectively. (**a**) qRT-PCR showed the relative RNA expression of KCNQ1OT1, miR-421-3p, mTOR, Col-1, RUNX2 and OCN of MC3T3-E1 cells. (**b**, **c**) Protein expression levels of pmTOR, mTOR, Col-1, RUNX2 and OCN of MC3T3-E1 cells after transfection and culture in osteogenic differentiation induction medium for seven days. Uncropped Western blot images are shown in Supplementary Fig. 8. (**d**) Transwell migration assay showed the migration ability of MC3T3-E1 cells among different transfection group. (**e**) CCK-8 assay showed the capacity of MC3T3-E1 cells to proliferate in various transfection groups. ns: no significant, **P < 0.01, ***P < 0.001.

Transfection of si-KCNQ1OT1 inhibited the mRNA and protein expression of pmTOR, mTOR, Col-1, RUNX2 and OCN, while the inhibitor of miR-421-3p rescued this effect (Fig. 6a–c). IF staining of RUNX2 and mTOR were also conformed to the above results (Supplementary Fig. 4a, b). Therefore, these experiments provide convincing evidence that lncRNA KCNQ1OT1 enhances osteogenic differentiation via affecting miR-421-3p/mTOR axis. Moreover, the inhibition of MC3T3-E1 migration and proliferation by si-KCNQ1OT1 was also reversed by the inhibitor of miR-421-3p (Fig. 6d,e).

### Expression levels of KCNQ1OT1, miR-421-3p, mTOR, and osteogenesis-related markers during osteogenic differentiation in vitro

**Figure 7:**
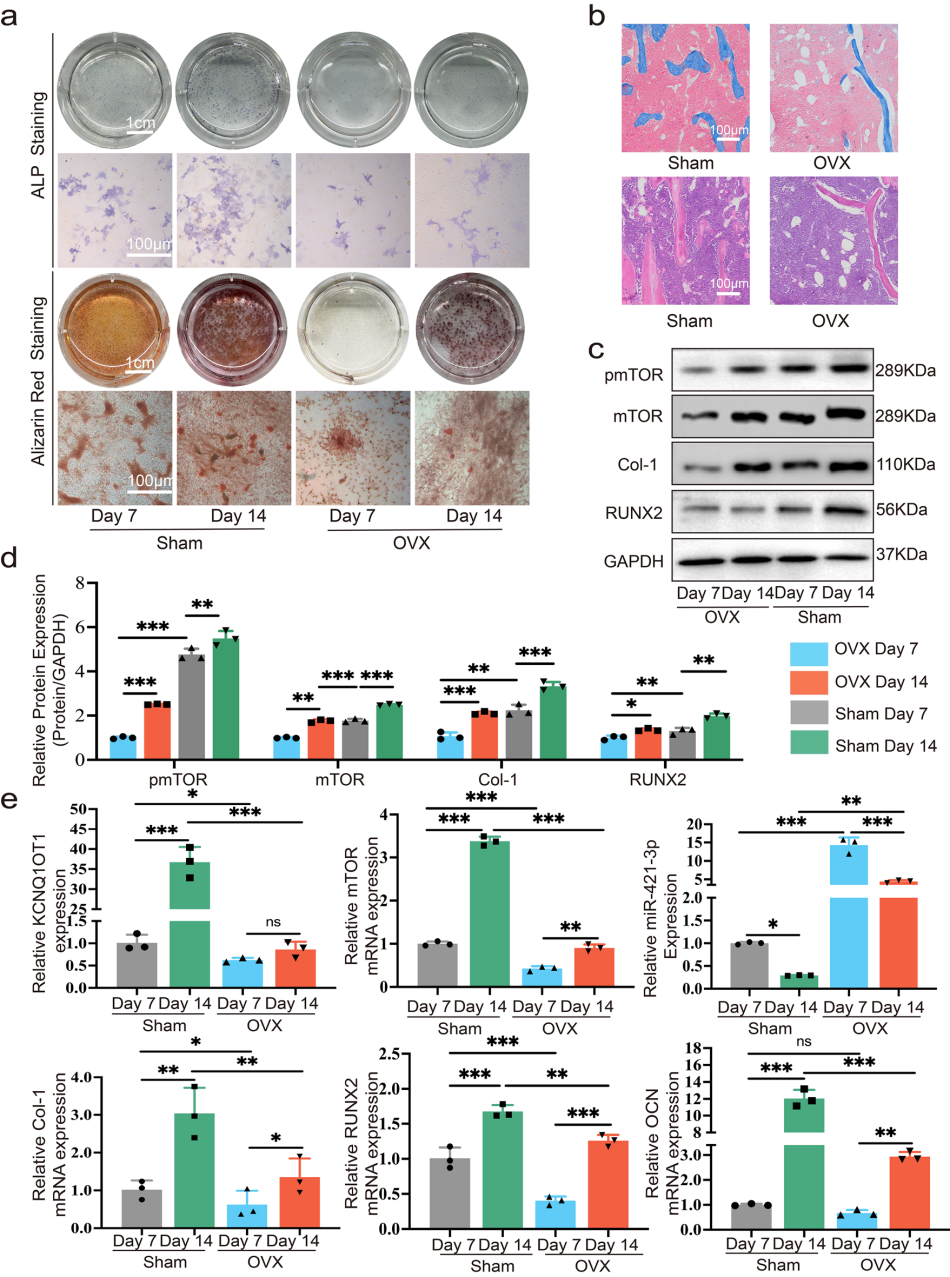
The expression levels of KCNQ1OT1, miR-421-3p, mTOR, and osteogenesis-related markers during osteogenic differentiation in vitro. (**a**) Osteoblasts in sham-operated and OVX groups were isolated and grown in a condition that induces osteogenic differentiation for 7 and 14 days, ALP staining and Alizarin Red staining were used to showed the degree of osteogenic differentiation. (**b**) Masson and HE staining of femurs from both sham and OVX group mice. (**c**, **d**) Protein expression levels of pmTOR, mTOR, Col-1 and RUNX2 of osteoblasts were detected by western blot after isolated and cultured in a condition that induces osteogenic differentiation for seven days and 14 days. Uncropped Western blot images are shown in Supplementary Fig. 9. (**e**) The RNA expression of KCNQ1OT1, miR-421-3p, mTOR, Col-1, RUNX2 and OCN of osteoblasts isolated from sham and OVX mice femurs and induction in vitro for seven and 14 days was detected by qRT-PCR. *P < 0.05, **P < 0.01, ***P < 0.001.

Three C57BL/6 mice were taken from each of the sham-operated and ovariectomy induced osteoporosis mouse model (OVX) groups. After euthanizing mice, their femurs were isolated, and osteoblast precursor cells were extracted and cultured. Then we examined changes in the relative expression of KCNQ1OT1, miR-421-3p, and mTOR after induction of osteogenic differentiation in MC3T3-E1 cells. We assessed the degree of MC3T3-E1 cells’ osteogenic differentiation through the dynamic changes of osteogenesis-related markers Col-1, RUNX2, and OCN, and we compared them with the expression changes of KCNQ1OT1/miR-421-3p/mTOR. Comparing the sham-operated group and the OVX group, the ALP levels in ALP staining and mineralized nodules in Alizarin red staining for the osteoblasts extracted from the sham-operated group on day seven were higher than those of the OVX group. The results at day 14 were the same, indicating that, compared with the Sham-operated group, PMOP induced by OVX led to a weakened osteogenic differentiation ability of mouse osteoblasts (Fig. 7a). The densities of collagen and trabecular bone were also significantly higher in the sham-operated group than in the OVX group (Fig. 7b). Interestingly, we found that the expression levels of KCNQ1OT1, mTOR, and pmTOR increased after induction of osteogenic differentiation. These results matched the expression level trends of osteogenesis-related markers Col-1, RUNX2, and OCN (Fig. 7c–e). However, the expression levels of miR-421-3p were decreased, in contrast to the trend of osteogenesis-related markers. The above results prove that the expressions of KCNQ1OT1 and mTOR in osteoblasts with strong osteogenic ability (i.e., osteoblasts of sham-operated group) were higher than in those with weak osteogenic ability (i.e., osteoblasts of OVX group); meanwhile, the expression of miR-421-3p was lower, indicating that KCNQ1OT1 and mTOR promoted osteogenic differentiation, while miR-421-3p inhibited osteogenic differentiation.

### Up-regulation of KCNQ1OT1 alleviates osteoporosis

**Figure 8:**
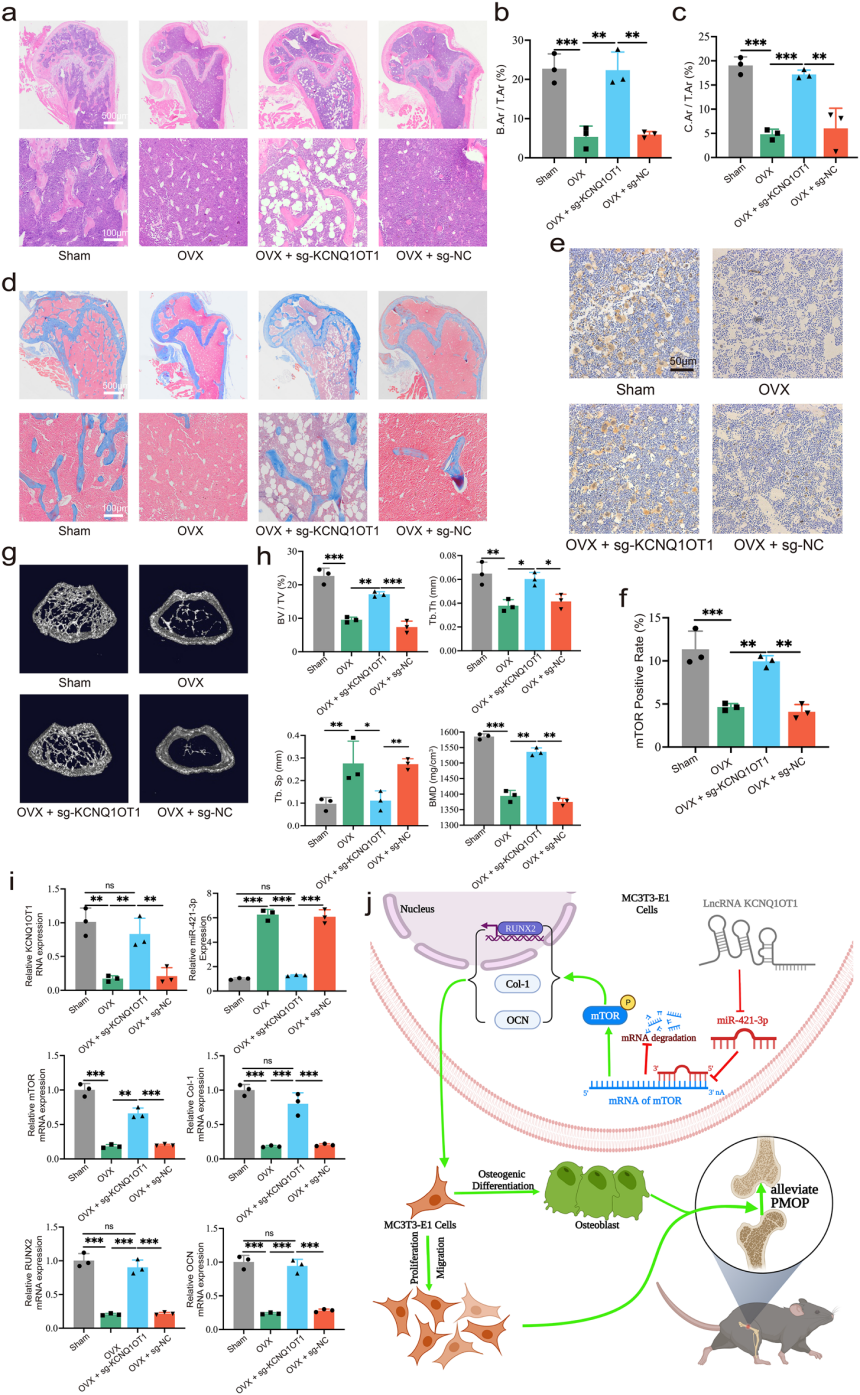
KCNQ1OT1 alleviates osteoporosis. Four groups of mice are as follows: (1) Sham, (2) OVX, (3) OVX + sg-KCNQ1OT1 AND (4) ovx + sg-NC (N = 3). (**a**, **b**) Each group's mice distal femurs were stained with HE and bone area/total area (B.Ar/T.Ar) was analyzed via images of HE staining. (**c**, **d**) Each group's mice distal femurs were stained with Masson and collagen area/total area (C.Ar/T.Ar) was analyzed via images of Masson staining. (**e**) IHC of mice femurs of each group. (**f**) mTOR positive rate was analyzed and calculated by IHC images of mice femurs. (**g**) Three-dimensional reconstruction of images from micro-CT of distal femurs from each group. (**h**) Parameters including bone mineral density (BMD), trabecular bone volume/tissue volume (BV/TV), trabecular separation (Tb.Sp) and trabecular thickness (Tb.Th) were analyzed by micro-CT. (**i**) Relative RNA expression of KCNQ1OT1, miR-421-3p, mTOR, Col-1, RUNX2 and OCN of mice femurs was performed by qRT-PCR. (**j**) KCNQ1OT1 sponges miR-421-3p to positively regulate mTOR expression at the post-transcriptional level, thereby enhances MC3T3-E1 cell function on proliferation, migration, and osteogenic differentiation in vitro and alleviates PMOP in vivo. *P < 0.05, **P < 0.01, ***P < 0.001.

OVX and sham groups of mice were treated differently. The H&E staining results showed that the bone area/total area ratio (B.Ar/T.Ar) of OVX mice was obviously decreased, when compared with sham-operated group mice. However, administration of sg-KCNQ1OT1 significantly recovered B.Ar/T.Ar (Fig. 8a,b). As for the Masson staining, the collagen area/total area ratio (C.Ar/T.Ar) of OVX mice was decreased, but administration of sg-KCNQ1OT1 restored C.Ar/T.Ar (Fig. 8c,d). mTOR IHC staining demonstrated that the positive rate of mTOR was greatly decreased in the OVX and OVX + sg-NC groups, whereas this trend was rescued by administration of sg-KCNQ1OT1 (Fig. 8e,f). In addition, we further investigated the effect of KCNQ1OT1 on bone structure. Three-dimensional reconstruction of images from micro-CT of distal femurs showed that the trabecular structure remained intact in the sham-operated group. However, trabecular structures were severely lost in the OVX and OVX + sg-NC groups, while administration of sg-KCNQ1OT1 mostly rescued this phenomenon (Fig. 8g). Micro-CT analysis showed significant decreases of trabecular bone volume/tissue volume (BV/TV), trabecular thickness (Tb.Th), trabecular separation (Tb.Sp) and bone mineral density (BMD) in the OVX and OVX + sg-NC groups, compared with the sham-operated group, while these decreases were reversed in the OVX + sg-KCNQ1OT1 group. Furthermore, the increases of Tb.Sp in the OVX and OVX + sg-NC groups were rescued by treatment with sg-KCNQ1OT1 (Fig. 8h). RNA was extracted from femoral bone tissue of mice in each group and qRT-PCR was performed. The results showed that the expression of KCNQ1OT1 in OVX mice injected with sg-KCNQ1OT1 was significantly higher than OVX mice treated with sg-NC or normal saline, but a bit lower than that in the sham-operated group (Fig. 8i). The expression levels of mTOR, RUNX2, Col-1 and OCN showed the same trend as KCNQ1OT1 (Fig. 8i), whereas the expression of miR-421-3p was the opposite. These results proved that up-regulation of lncRNA KCNQ1OT1 could alleviate PMOP in OVX mice through the miR-421-3p/mTOR axis.

## Discussion

Females are more prone to OP after menopause due to a decline in estrogen levels. As such, the prevalence of OP is much higher in females than in males^1^. At the same time, the severity of OP in PMOP patients is also heavier than that caused by other etiologies^33^. Therefore, PMOP patients are more prone to fragility fractures, which pose a serious threat to quality of life and health^34^. At present, many drugs aimed at stimulating osteoblast activity and inhibiting osteoclasts are available^35^; however, there are not many drugs that can stimulate the activity of osteoblasts, and the commonly used active vitamin K2 and teriparatide require frequent use of medication and long treatment courses, resulting in poor patient compliance, which leads to poor treatment effect^36^. Thus, it is necessary to explore new mechanisms by which osteoblast activity can be stimulated, in order to provide new targets for PMOP treatment. In this study, we mainly focused on PMOP, the most common type of OP^37^.

We simulated PMOP using an OVX-induced PMOP mouse model^38^. And we demonstrated, for the first time, that injection of lncRNA KCNQ1OT1 over-expression virus could promote trabecular bone remodeling and collagen regeneration in OVX mice, thereby alleviating PMOP. Simultaneously, we demonstrated that over-expression of KCNQ1OT1 up-regulated mTOR expression in the femurs of OVX mice. We also extracted osteoblast precursor cells from the femurs of OVX and sham-operated mice to induce osteogenic differentiation in vitro^39^. We discovered that RUNX2, Col-1, and OCN all had greater levels of expression in the sham-operated control group than in the OVX group, and the expression of the above osteogenesis-related markers also increased with the prolongation of induction time. Interestingly, the expression of KCNQ1OT1 showed the same trend, indicating that the expression of KCNQ1OT1 elevated with the enhancement of the osteogenic differentiation ability. In addition, KCNQ1OT1 expression in PMOP patients was lower than in controls without PMOP. Previous studies have shown that KCNQ1OT1 can promote the osteogenic differentiation of hBMSCs and mMSCs, as well as inhibiting the apoptosis of MC3T3-E1 cells^40,41^. We found that KCNQ1OT1 can elevate the proliferation ability of MC3T3-E1 cells; in depth, it could promote MC3T3-E1 cells to the S phase of mitosis, thereby enhancing mitosis and proliferation. KCNQ1OT1 also enhances the migration ability of MC3T3-E1 cells. In addition, KCNQ1OT1 enhanced the osteogenic differentiation of MC3T3-E1 cells via promoting the expression of RUNX2, a key transcription factor regulating osteogenic differentiation, and then up-regulated the expression of Col-1 and OCN. Taken together, the evidence above proves that KCNQ1OT1 can enhance the proliferation, migration, and osteogenic differentiation of MC3T3-E1 cells in vitro, thus rescuing PMOP in vivo.

Using Starbase, Targetscan, and miRwalk, we discovered that miR-421-3p was a putative KCNQ1OT1 target which may also regulate mTOR expression. More and more studies have shown that cytoplasmic lncRNAs can sponge miRNAs^42–45^. In this regard, we determined the localization of lncRNA KCNQ1OT1 through RNA-FISH, through which we found that KCNQ1OT1 was mainly located in the cytoplasm, which aligned with other published research, and we found that miR-421-3p was also located in the cytoplasm. Dual luciferase reporter assays demonstrated the targeting relationships of KCNQ1OT1 and miR-421-3p, as well as miR-421-3p and mTOR. RIP assay further certified that KCNQ1OT1 interacted directly with miR-421-3p. Therefore, we predicted that the KCNQ1OT1/miR-421-3p/mTOR axis might be involved in the regulation of MC3T3-E1 cells proliferation, migration, and osteogenic differentiation. Previous studies have shown that the osteosarcoma cell lines MG63 and U2OS migrated and invaded more readily when miR-421 was over-expressed^46,47^. There has been no study verifying the regulatory effect of miR-421-3p on MC3T3-E1 cells. We found that, compared with the NC group, the up-regulation (via mimic) of miR-421-3p caused MC3T3-E1 cells to arrest in the G1 phase of mitosis and inhibited their proliferation, while the down-regulation (via inhibitor) of miR-421-3p promoted MC3T3-E1 cells to G2 and S phases, thereby enhancing the proliferation of cells. miR-421-3p also suppressed the migration ability of MC3T3-E1 cells. Moreover, we identified that miR-421-3p suppressed osteogenic differentiation and decreased the expression of osteogenesis-related markers. We also proved that the expression of miR-421-3p was elevated significantly in PMOP patients and osteoblast precursor cells extracted from OVX mice.

Previous studies have demonstrated that mTOR could enhance the osteogenic differentiation of BMSCs and MC3T3-E1 cells^48,49^. Dual luciferase assay proved that miR-421-3p targets mTOR. As in previous studies, we observed that down-regulation (via siRNA) of mTOR inhibited the osteogenic differentiation of MC3T3-E1 cells. Furthermore, we found that si-mTOR suppressed the proliferation and migration of MC3T3-E1 cells, while the miR-421-3p inhibitor significantly reversed the effects of si-mTOR. In addition, the expression level of mTOR in PMOP patients was lower than those without PMOP, similar to the results in osteoblasts of OVX and sham-operated mice. Furthermore, our findings unequivocally demonstrated that KCNQ1OT1 might regulate mTOR by contendingly binding to miR-421-3p and suppressing its function. Over-expression of KCNQ1OT1 promoted the expression of mTOR. In addition, the miR-421-3p inhibitor obviously rescued the suppressive effects of si-KCNQ1OT1 and si-mTOR on the proliferation, migration, and osteogenic differentiation of MC3T3-E1 cells. Overall, the role of the KCNQ1OT1/miR-421-3p/mTOR axis in regulation of the proliferation, migration, and osteogenic differentiation of MC3T3-E1 cells was discovered and confirmed in vitro and vivo. However, further research is required, in order to explore how the KCNQ1OT1/miR-421-3p/mTOR axis affects the mTORC1 and mTORC2 pathways, respectively, and whether it affects other osteogenesis-related signaling pathways.

In conclusion, in this study, we proved that KCNQ1OT1 could enhance proliferation, migration, and osteogenic differentiation of MC3T3-E1 cells. KCNQ1OT1 alleviated PMOP in an OVX mouse model by acting as a miR-421-3p sponge to upregulate mTOR (Fig. 8j). This proven regulatory axis could provide new markers for OP diagnosis, and could facilitate the development of more effective treatments for OP.

## Materials and methods

### Clinical samples

Ten female inpatients who underwent lumbar surgery at the Qilu Hospital from August 2021 to October 2021 were selected. The BMD of participants was measured by dual-energy X-Ray absorptiometry (DEXA) before surgery was performed. A lumbar T score less than − 2.5 was diagnosed as OP. Among the ten participants, five patients were diagnosed with OP (62.2 ± 5.42 years), while BMDs of the other five patients were normal (62.8 ± 5.34 years). A total of 0.5 g cancellous bone for each patient was collected from the surgically removed lumbar bone tissue. This study was authorized and approved by the Qilu Hospital ethics committee of Shandong University. And the Declaration of Helsinki was followed for the conduct of this study. All participants were aware of this research and gave their informed consent.

### Bioinformatic analysis

Starbase 2.0 was used for predicting miRNAs that sponge with lncRNA KCNQ1OT1. The miRNAs that might regulate mTOR were forecasted using Starbase 2.0, Targetscan and miRwalk. The information from the above databases was displayed via Venn diagrams, and the intersections showed the miRNAs of interest.

### Cell culture

HEK-293T cells and mouse pre-osteoblast MC3T3-E1 cells were obtained from the Chinese Academy of Sciences Cell Bank (Shanghai, China). The HEK-293T cells were cultivated in DMEM (Gibco, USA) supplemented with 1% penicillin/streptomycin (Gibco, USA) and 10% fetal bovine serum (FBS, Gibco, USA). MC3T3-E1 cells were cultured in α-MEM (Gibco, USA) mixed with 1% penicillin/streptomycin (Gibco, USA) and 10% FBS (Gibco, USA). All cell lines were cultured under conditions of 5% CO_2_ and 95% humidity, and they were not used beyond 20 generations. In order to study the osteogenic differentiation, osteogenesis induction medium containing 10 mM β-glycerophosphate (Sigma, USA), 50 μM ascorbic acid (Solarbio, China) and 100 nM dexamethasone (Solarbio, China) was used to culture cells.

### Cell transfection

The sg-KCNQ1OT1 and sg-NC were fabricated by using the clustered regularly interspaced short palindromic repeat (CRISPR)-associated protein 9 synergic activation mediator (CRISPR/Cas 9 SAM) technology of Genechem (Shanghai, China). Above vectors were transfected into cells as instructed by the manufacturer. Si-KCNQ1OT1, si-mTOR, and mimic and inhibitor of miR-421-3p with their NCs were designed and produced by GenePharma (Shanghai, China). MC3T3-E1 cells were transfected with the aforementioned siRNAs and miRNAs using the lipofectamine 2000 transfection agent (Invitrogen, USA).

### Quantitative real-time polymerase chain reaction (qRT-PCR)

TRIzol (Thermo Fisher Scientific, USA) was used to extract the total RNA from bone tissues or cells, after which RNA was quantified through a NanoDrop spectrophotometer (Thermo Scientific, Germany). Then, 1000 nanograms of RNA were reverse transcribed to cDNA using a ReverTra Ace qPCR RT Kit (for lncRNAs and mRNAs, TOYOBO, Japan) or a U6 snRNA Real-time RT-PCR Kit (for miRNA, GenePharma, China). SYBR® Green Realtime PCR Master Mix (for cDNA of mRNAs and lncRNA, TOYOBO, Japan) and a U6 snRNA Real-time RT-PCR Kit (for cDNA of miRNA, GenePharma, China) were applied to complete qRT-PCR with a CFX96 Real-time System (BIO-RAD, USA). Gene expression quantification was analyzed through the 2^−ΔΔCt^ method, with GAPDH and U6 normalized mRNA and miRNA expression. The forward and reverse primers used for qRT-PCR are displayed in Suppl Table S1.

### Western blot

Cells seeded in wells were lysed in an ice-cold mixture of PMSF and RIPA lysis buffer (Beyotime, China), following which they were centrifuged at 12,000 rpm for 15 min. A BCA protein assay kit (Beyotime, China) was used to detected the concentration of the protein samples. The same amount of protein was loaded and separated by SDS-PAGE, transferred to polyvinylidene difluoride (PVDF) membranes, blocked for 1 h with 5% nonfat milk and then soaked with primary antibodies against phospho-mTOR (pmTOR, 1:1000, Cell Signaling Technology #5536, USA), mTOR (1:1000, Cell Signaling Technology #2983, USA), Collagen-1 (Col-1, 1:1000, Abcam ab138492, UK), RUNX2 (1:1000, Cell Signaling Technology #12556s, USA), Osteocalcin (OCN, 1:1000, Abcam ab93876, UK) and GAPDH (1:1000, Cell Signaling Technology #5174, USA) at 4 °C in a shaker overnight. After that, HRP-conjugated secondary antibody (1:5000, Cell Signaling Technology, #7074, USA) was incubated with membranes at room temperature for 1 h. Eventually, protein levels were observed using Chemiluminescent HRP Substrate (Millipore, Germany) and analyzed using the ImageJ software.

### Cell counting kit 8 (CCK-8) assay

MC3T3-E1 cells were seeded in 96-well plates (2 × 10^3^ cells per well). After transfection, the optical density (OD) value at 450 nm, representing cell proliferation ability, was detected at 0 h, 24 h, 48 h, and 72 h. 10 μL CCK-8 solution (Elabscience, China) was added into each well and was incubated at 37 °C in the dark for two hours. The OD value was recorded by a microplate reader (BioTek, USA) using 100 μL of solution in a 96-well plate in triplicate.

### Transwell migration assay

Cells (2 × 10^4^ cells per well) were resuspended in FBS-free medium and added to the upper chambers of Transwell plates (Corning, USA), while 700 μL normal culture medium (including 10% FBS as an inducer) was placed in the wells of 24-well plate. After incubation for 24 h, cells on the upper chambers were removed, and cells which migrated to the lower surface of filter membranes were immobilized with methanol, washed with PBS, and stained with 0.1% crystal violet (Solarbio, China). Taking pictures of migrated cells under a microscope's high-power field (HPF), cell migration ability was calculated with respect to the average number of MC3T3-E1 cells per HPF.

### Cell cycle assay

After transfection of MC3T3-E1 cells for 72 h, a Cell Cycle Assay Kit (BestBio, China) was used to detect the cell cycle. Cells were held in 75% ethanol for one hour at − 20 °C. Then, cells were centrifuged and resuspended in ice-cold PBS containing 1% BSA. Subsequently, MC3T3-E1 cells were incubated with RNAse A solution for 30 min at 37 °C and then incubated with propidium iodide (PI) for one hour at 4 °C. Finally, cell cycle was performed by flow cytometry (Cytoflex S, Beckman Coulter, USA), using CytoExpert and FlowJo software.

### RNA immunoprecipitation (RIP) assay

The RIP assay was performed using a Magna RIP RNA-Binding Protein Immunoprecipitation Kit (Millipore, USA). Briefly, after being lysed, the lysate was treated with RIP buffer (Millipore, USA) containing magnetic beads coupled with either a negative control IgG or an anti-Ago2 antibody. After the antibody was recovered using protein A/G beads, qRT-PCR was carried out to determine whether KCNQ1OT1 and miR-421-3p were enriched in the precipitates.

### RNA fluorescent in situ hybridization (RNA-FISH) assay

Cy3-labeled KCNQ1OT1 and FAM-labeled miR-421-3p probes were designed and produced by GenePharma (Shanghai, China). RNA-FISH was performed using an RNA FISH SA-Biotin Amplification System Kit (GenePharma, China) according to the manufacturer's instructions. Nuclei were stained using DAPI. Finally, using the Spinning Disc Laser Confocal Microscope (Dragonfly 200, Oxford Instruments Andor, USA), it was possible to determine the locations of the lncRNA KCNQ1OT1 and the miR-421-3p in MC3T3-E1 cells.

### Dual luciferase reporter assay

Dual luciferase reporter gene constructs (KCNQ1OT1 WT, KCNQ1OT1 mut or mTOR WT, mTOR mut) and miR-421-3p mimic or NC were co-transfected into HEK-293T cells. After 24 h, the luciferase activity was measured using a Dual Luciferase Reporter Assay System (Promega, USA).

### Immunofluorescence (IF)

A total of 3 × 10^4^ MC3T3-E1 cells or stable virus-transfected strain were seeded on each glass coverslip (Solarbio, China). After 72 h of transfection, cells were fixed with 4% paraformaldehyde before being permeabilized for 20 min with 0.2% Triton-X 100 (Solarbio, China), blocked for 30 min with goat serum (Booster, China), and incubated with primary antibodies against mTOR (1:1000, Cell Signaling Technology #2983, USA) or RUNX2 (1:1000, Cell Signaling Technology #12556s, USA) overnight at 4 °C. Then, they were incubated with secondary antibody DyLight 488 (1:100, Abbkine #A23220, USA) for one hour. In addition, the nuclei were stained with DAPI, and actin was stained with TRITC-phalloidin (Solarbio, China). Finally, samples were observed by a Spinning Disc Laser Confocal Microscope (Dragonfly 200, Oxford Instruments Andor, USA). Under the condition that the shooting parameters were unchanged, the fluorescence intensity of related proteins was used to indicate the relative expression of protein in the cells.

### Alizarin red staining

At 14 days after transfection (MC3T3-E1 cells extracted from mouse femurs stained at seven and 14 days) and culturing in osteogenesis induction medium, 4% paraformaldehyde was used to fixed cells and Alizarin red (Beyotim, China) was used to stain calcified nodules. The results were examined using a microscope (Leica, Germany) after PBS washes.

### Alkaline phosphatase (ALP) staining

Seven days after transfection (MC3T3-E1 cells isolated from mouse femurs stained at seven and 14 days) and culturing in osteogenesis induction medium, an NBT/BCIP staining kit (Beyotime, China) was used to stain MC3T3-E1 cells according to the protocol of manufacturer. The images were acquired using a microscope (Leica, Germany).

### Animal ovariectomy (OVX)-induced osteoporosis model

All animal experiments obeyed the Animal Research: Reporting of In Vivo Experiments (ARRIVE) guidelines (Suppl 3), and the Animal Ethics Committee of Qilu Hospital approved them. A total of 40 female C57BL/6 mice (12 weeks old, mean weight of 20.4 g) were purchased from the Charles River Laboratory Animal Company (Beijing, China). The two groups of mice–one with a bilateral ovariectomy (OVX, 30 mice) and the other with a sham operation (Sham, 10 mice)–were chosen at random. The mice were placed inside the isoflurance chamber of the Isoflurance Veterinary Anesthesia System (PerkinElmer, Japan) until they were immobilized. After immobilization, mice were placed in a prone position and connected with an isoflurane nose cone. Double dorsolateral incisions were used, a muscle tissue was blunt dissected. Then, the ovarian fat pad was found and pulled out of the incision, then knotted with thread under the ovary. Subsequently, the ovaries distal to the knots were resected bilaterally. The sham operation group used the same surgical incisions as the OVX. And after separating the ovarian fat pad, and identifying the ovary, the ovary was returned to the abdominal cavity. Both groups underwent meticulous suturing of the wounds. Mice were placed on a heating pad until they recovered. Before the next experiments, the two groups of mice were raised independently for eight weeks. Qilu Hospital of Shandong University Animal Ethics Committee approved the study procedures.

### Osteoblast isolation and culture from mice model

Three OVX mice and three sham-operated control mice were randomly selected. Then, the mice were euthanized and their femurs were aseptically isolated and collected. After the bone marrow was washed away with Hanks buffer (Sigma, USA), the bone marrow-free femurs were quickly shredded in new Hanks buffer, placed in a centrifuge tube, and the supernatant was discarded. Then, 0.25% trypsin was added at a ratio of 1:30 and incubated at 37 °C for 30 min. After centrifugation, the supernatant was discarded, 0.2% collagenase type II (Sigma, USA) was added at a ratio of 1:5, and the mixture was incubated for 18 min in a shaker at 37 °C and 120 rpm. This step was repeated five times. The precipitate obtained after the last centrifugation was added with five times the volume of α-MEM, and the liquid was filtered through a 200-mesh metal mesh. The filtrate was centrifuged and resuspended in α-MEM containing 10% FBS, then cultured. Osteoblasts were extracted from each mouse according to the above protocol, cultured to logarithmic growth phase, then inoculated into 6-well plates, and cultured in osteogenic differentiation induction medium for seven or 14 days. We compared various biological properties of osteoblasts from OVX mice and sham-operated mice via qRT-PCR, Western blot, alizarin red staining, and ALP staining.

### Treatment of model mice

Forty mice were randomly divided into four groups: (1) Sham-operated (Sham) mice, (2) OVX, (3) OVX + sg-KCNQ1OT1, and (4) OVX + sg-NC (N = 3). The OVX + sg-KCNQ1OT1 and OVX + sg-NC groups were injected with sg-KCNQ1OT1 and sg-NC on days 1, 7, 14, and 21, respectively. The other two groups were injected with normal saline on days 1, 7, 14, and 21. On day 28, mice were euthanized, and the bilateral femurs were isolated and obtained. To ensure statistical independence, only the right femur from each mouse was used for each experiment. Each femur, therefore, was considered to be an experimental unit. Qilu Hospital of Shandong University's Animal Ethics Committee authorized the study's procedures.

### Micro-CT analysis

After being fixed with 4% paraformaldehyde for 24 h, mouse femurs were scanned with a micro-CT imaging system (PerkinElmer, Japan) at 90 kV and 88 μA. Each femur was scanned 360º in high-resolution mode for 14 min. The region of interest (ROI) was the 4 × 4 × 4 mm^3^ area located 1 mm below the epiphyseal plate of the distal femur. Parameters such as bone mineral density (BMD), trabecular bone volume/tissue volume (BV/TV), trabecular thickness (Tb.Th), and trabecular separation (Tb.Sp) were analyzed using Analysis software (PerkinElmer, Japan).

### Histologic and immunohistochemistry (IHC) staining

Femurs were fixed in 4% paraformaldehyde, decalcified in 10% ethylenediaminetetraacetic acid (Servicebio, China), and embedded in paraffin. Then, samples were cut into sections with 4 μm thickness. For histological analysis, bone sections were stained with hematoxylin & eosin (H&E) or Masson according to the manufacturer’s constructions. For IHC analysis, bone sections were deparaffinized, rehydrated, treated with Antigen Retrieval Solution (Servicebio, China), blocked with BSA and incubated with primary antibody against mTOR (1:100, Cell Signaling Technology #2983, USA) overnight. Then secondary antibody was used to incubate with sections for one hour. Eventually, the coverslips were glued with Rhamsan gum (Servicebio, China). A microscope was used to capture images and imageJ software was used to analyze the mTOR positive area.

### Statistical analysis

Data obtained from experiments were analyzed with Prism 9.0 (GraphPad Software, USA) and were expressed as mean ± standard deviation (SD). The differences between more than two groups were analyzed by one-way analysis of variance (ANOVA). Student’s t-test was used to examine the differences between two groups. P < 0.05 was considered statistically significant. All experiments were repeated three times.

### Ethical approval

The experiments about human individuals were performed in accordance with the Declaration of Helsinki, and approved by the Qilu Hospital ethics committee of Shandong University. The animal experiments obeyed the Care and Use of Laboratory Animals Guidelines, and the Animal Ethics Committee of Qilu Hospital of Shandong University approved them.

## Supplementary Information

Supplementary Information 1.

Supplementary Information 2.

Supplementary Information 3.

## Data Availability

All data generated or analyzed during this study are included either in this article or in the supplementary files.
