# Supplementary material for: Long non-coding RNA KCNQ1OT1 alleviates postmenopausal osteoporosis by modulating miR-421-3p/mTOR axis
Source: Sci Rep. 2023 Feb 9;13:2333. doi: 10.1038/s41598-023-29546-4 (PMC9911397; doi:10.1038/s41598-023-29546-4)
Supplement: Supplementary file 1 — Supplementary Information 1. [file 41598_2023_29546_MOESM1_ESM.pdf]

Supplementary Figure for:

Long Non-coding RNA KCNQ1OT1 Alleviates Postmenopausal Osteoporosis by Modulating miR-421-3p/mTOR Axis

Ziyu Wang[1, 2], Hengshuo Zhang[1, 2], Qinghui Li[1, 2], Lu Zhang[1, 2],
Lu Chen[2], Hongliang Wang[1] and Yunzhen Chen[1,2,*]

[1]Department of Orthopedics, Qilu Hospital of Shandong University, Jinan 250012, PR China
[2]Cheeloo College of Medicine, Shandong University, Jinan 250012, PR China
*Corresponding Author: Yunzhen Chen

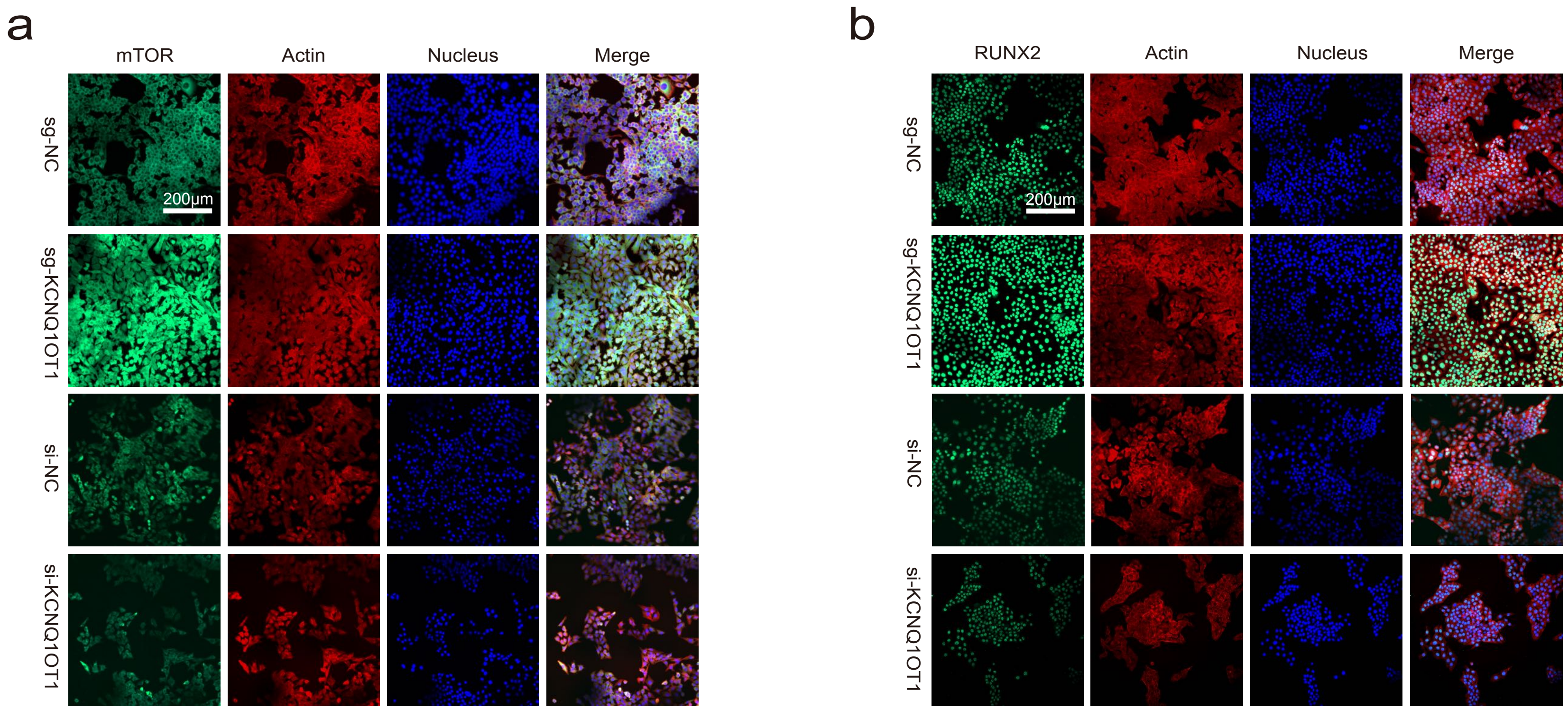

Supplementary Figure 1. Immunofluorescent staining of MC3T3-E1 cells after being transfected with sg-KCNQ1OT1, sg-NC, si-KCNQ1OT1, si-NC, respectively. (a) mTOR. (b) RUNX2.

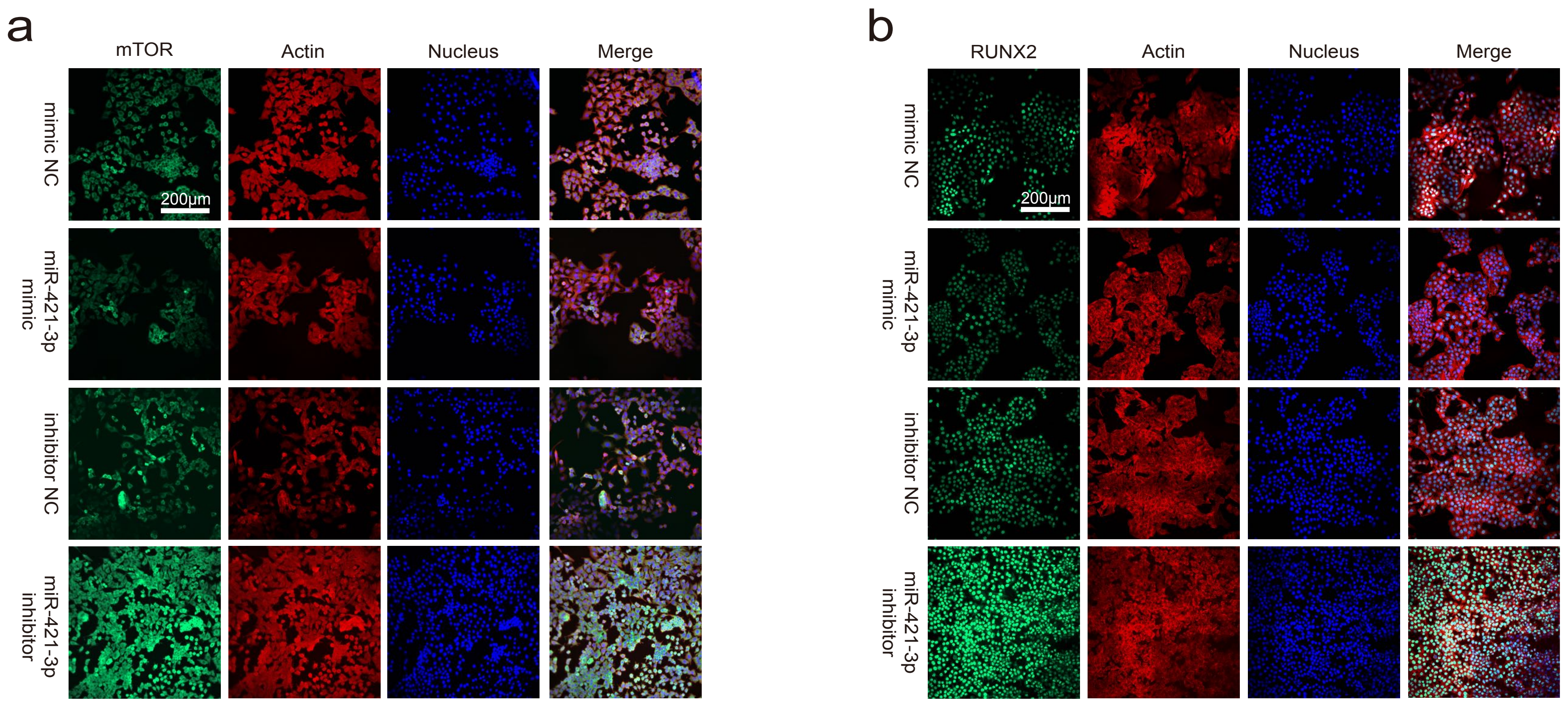

Supplementary Figure 2. Immunofluorescent staining of MC3T3-E1 cells after being transfected with miR-421-3p mimic, mimic NC, miR-421-3p inhibitor or inhibitor NC, respectively. (a) mTOR. (b) RUNX2.

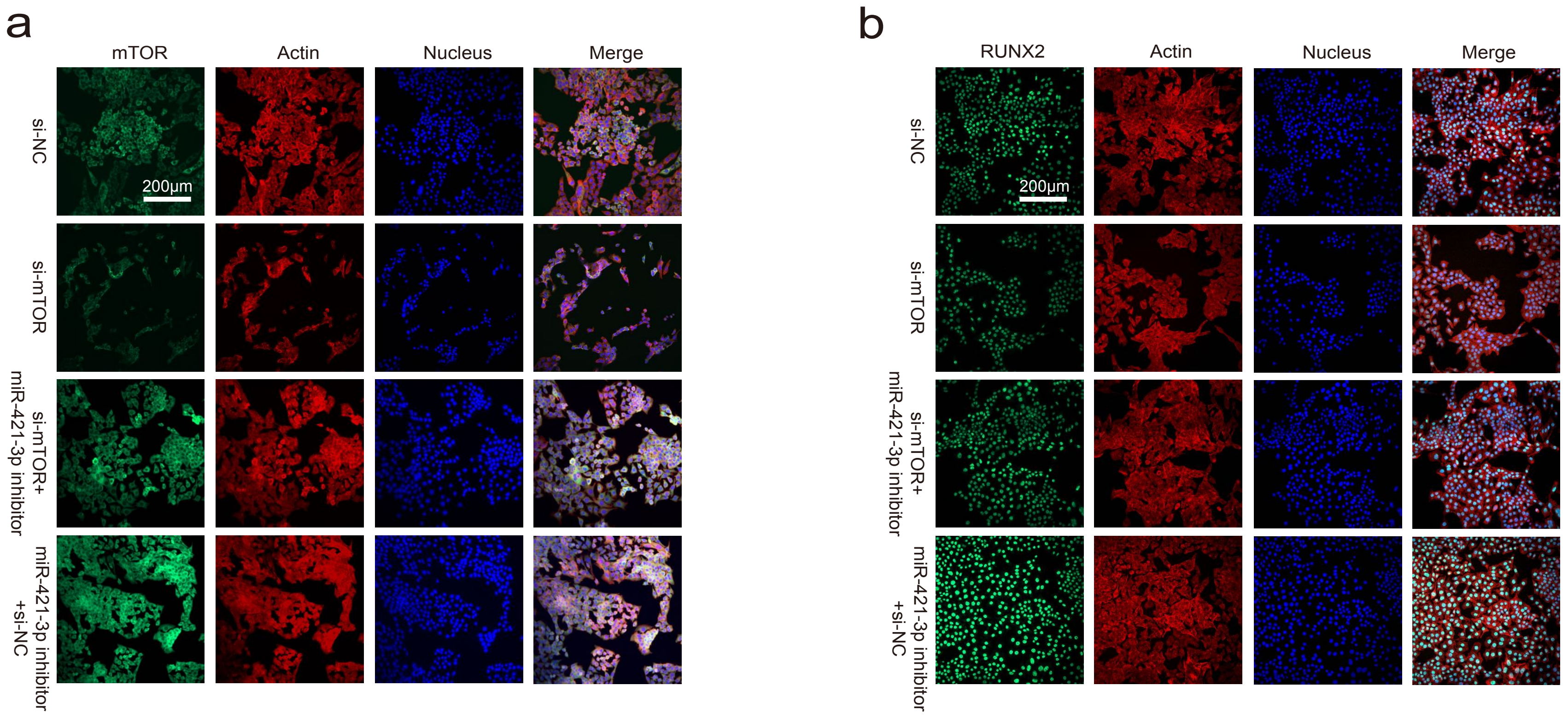

Supplementary Figure 3. Immunofluorescent staining of MC3T3-E1 cells after being transfected with si-mTOR, si-NC, si-mTOR + miR-421-3p inhibitor or si-NC + miR-421-3p inhibitor, respectively. (a) mTOR. (b) RUNX2.

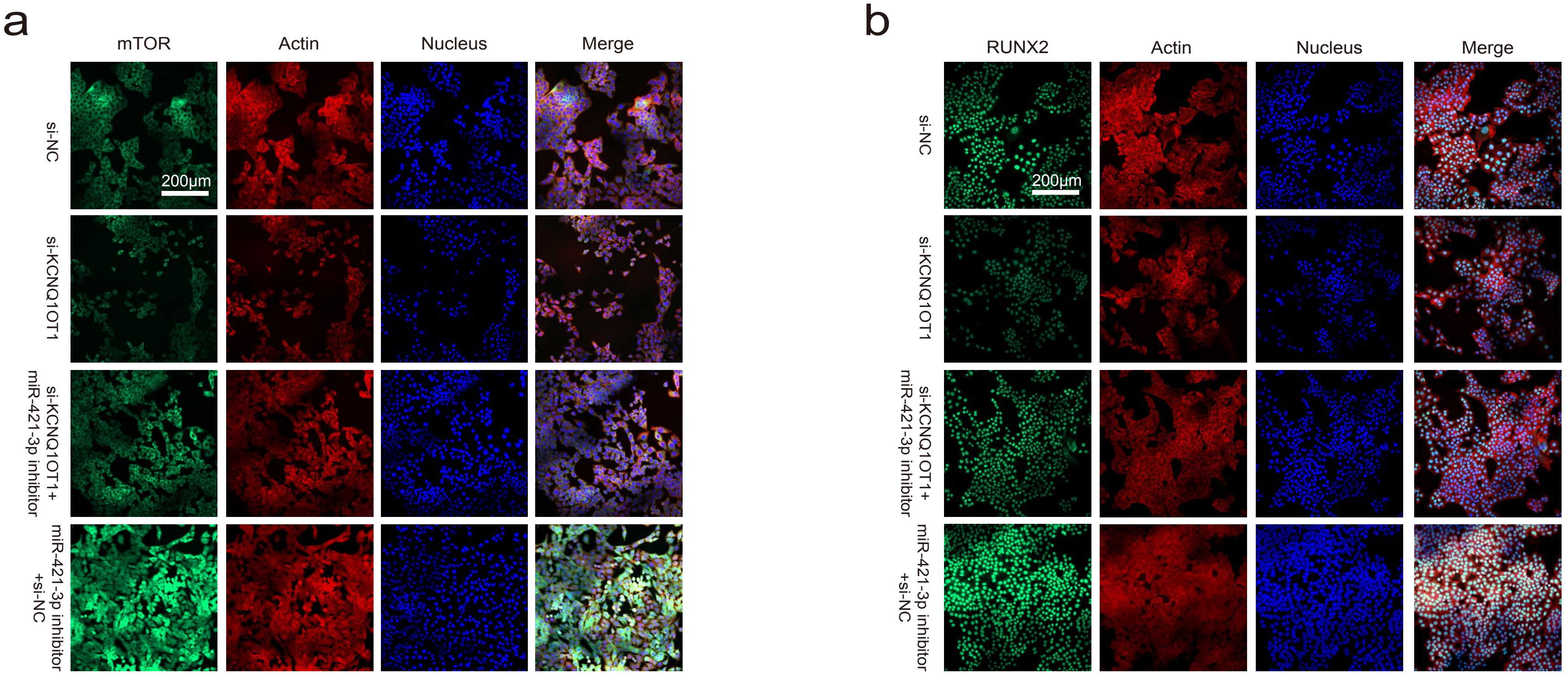

Supplementary Figure 4. Immunofluorescent staining of MC3T3-E1 cells after being transfected with si-KCNQ1OT1, si-NC, si-KCNQ1OT1 + miR-421-3p inhibitor or si-NC + miR-421-3p inhibitor, respectively. (a) mTOR. (b) RUNX2.

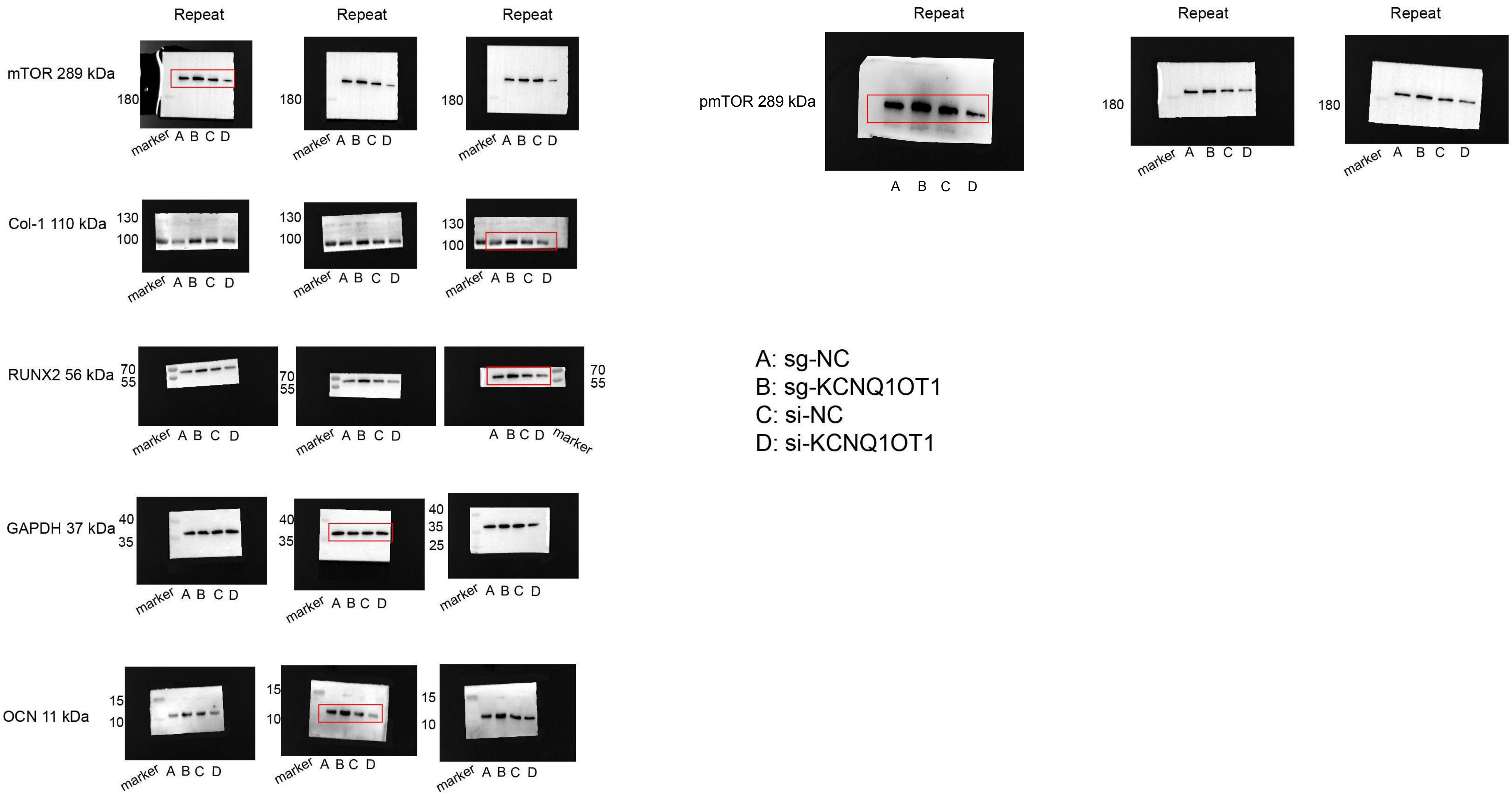

Supplementary Figure 5. Unprocessed western blots. Source Data for Figure 1h.

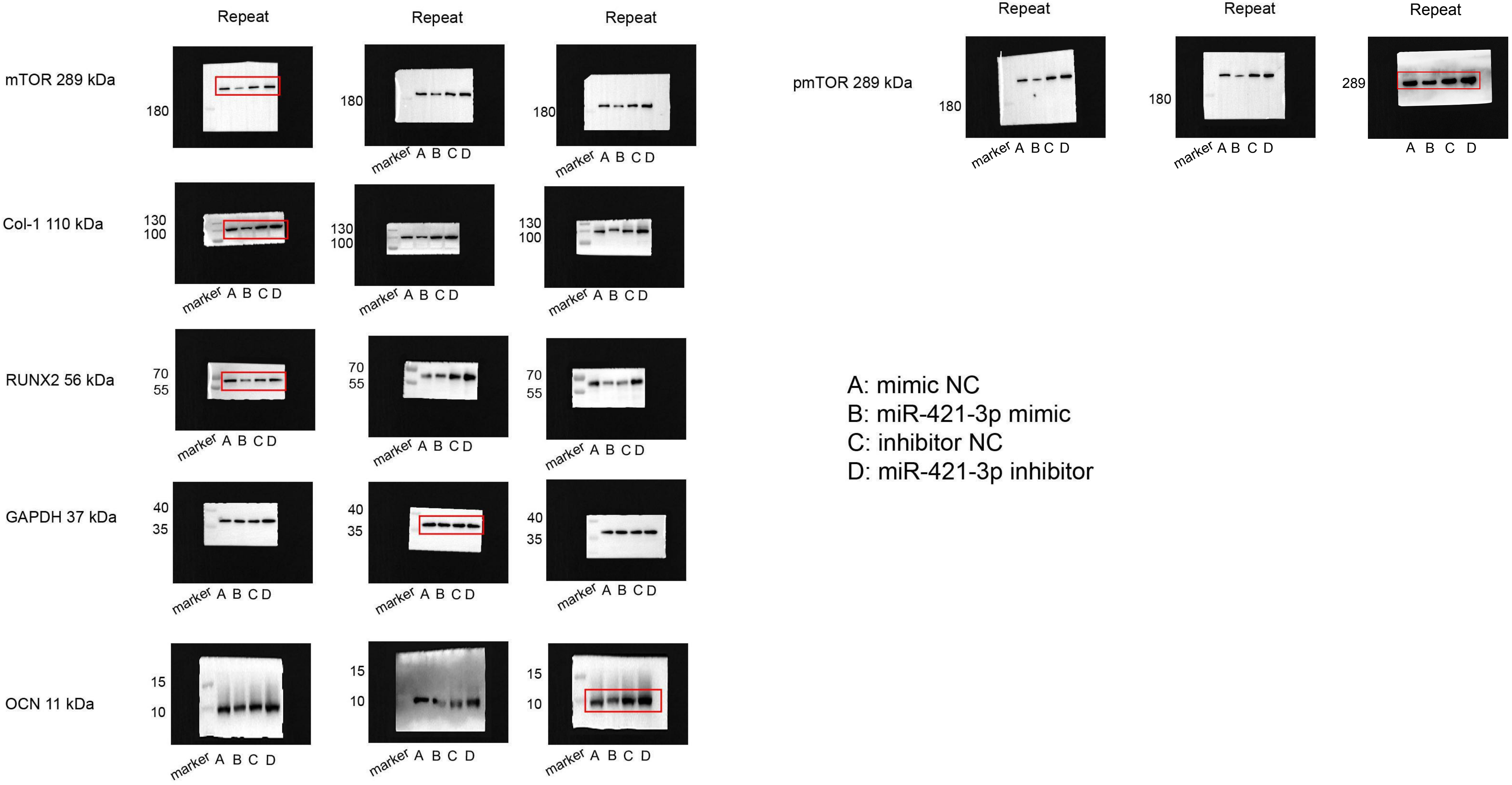

Supplementary Figure 6. Unprocessed western blots. Source Data for Figure 4c.

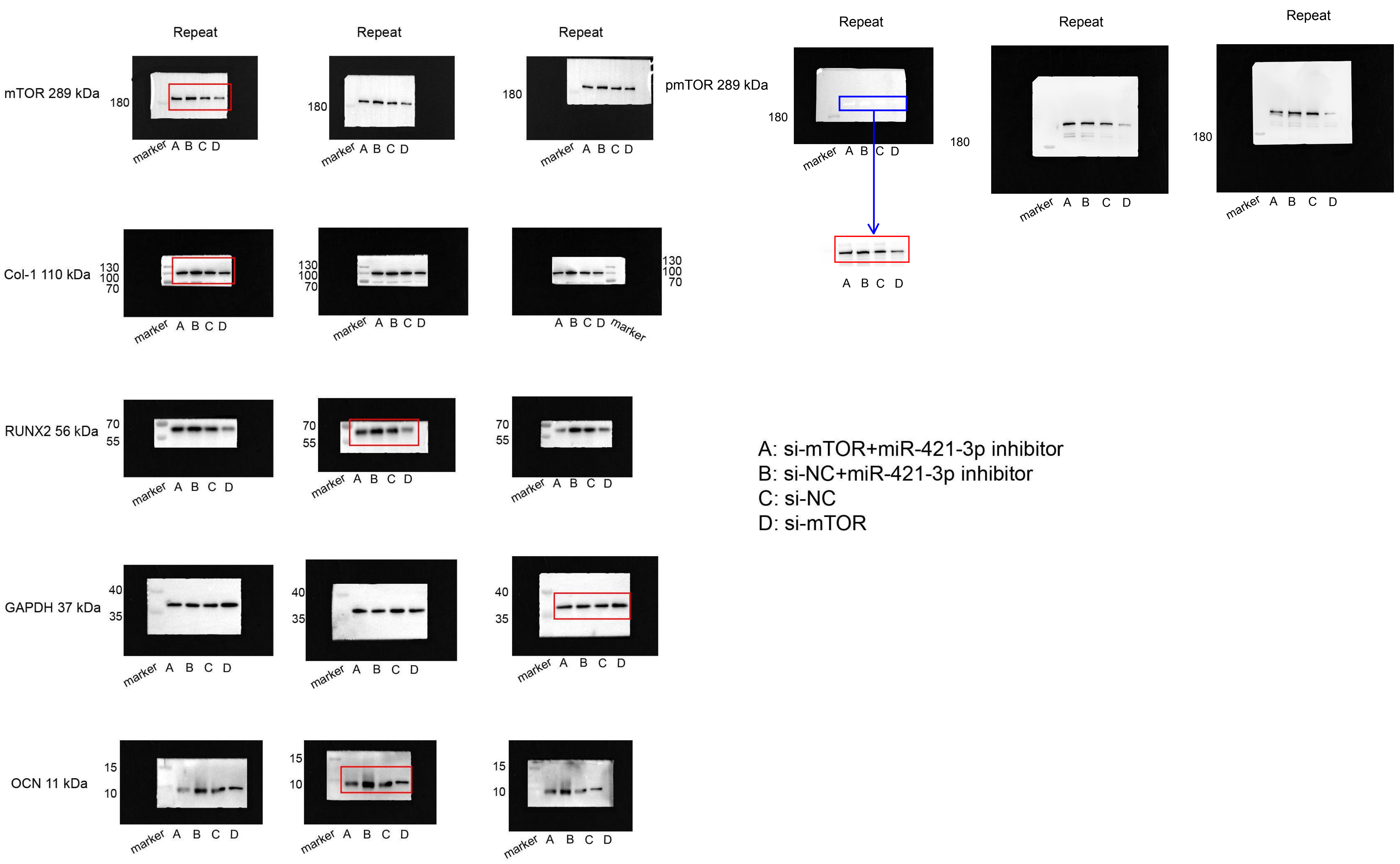

Supplementary Figure 7. Unprocessed western blots. Source Data for Figure 5f.

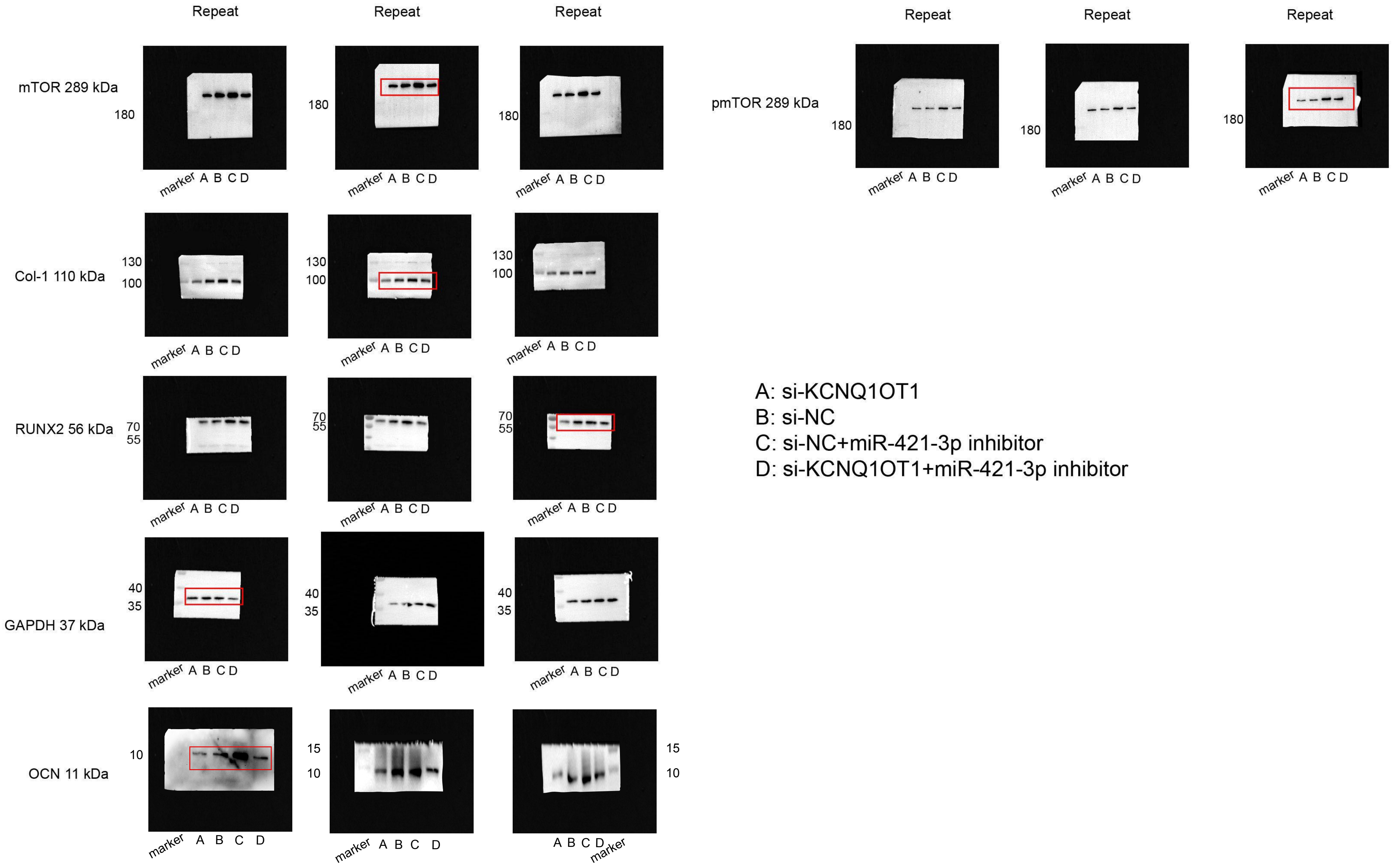

Supplementary Figure 8. Unprocessed western blots. Source Data for Figure 6b.

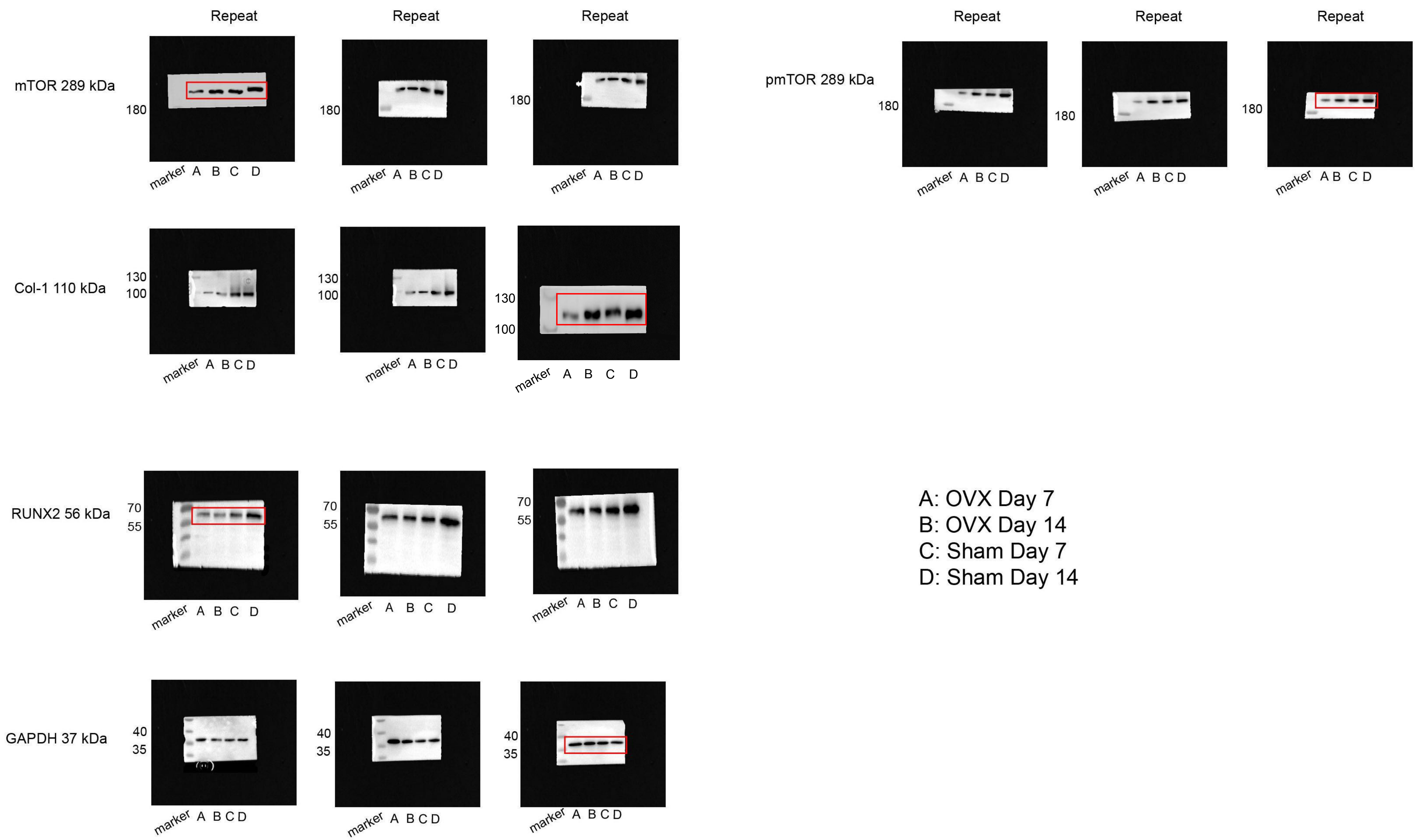

Supplementary Figure 9. Unprocessed western blots. Source Data for Figure 7c.
