# Supplementary material for: Long non-coding RNA KCNQ1OT1 alleviates postmenopausal osteoporosis by modulating miR-421-3p/mTOR axis
Source: Sci Rep. 2023 Feb 9;13:2333. doi: 10.1038/s41598-023-29546-4 (PMC9911397; doi:10.1038/s41598-023-29546-4)
Supplement: Supplementary file 2 — Supplementary Information 2. [file 41598_2023_29546_MOESM2_ESM.docx]

Table S1. Primers used in the qRT-PCR.

| Primer | Direction | Squences (5’- 3’) |
| --- | --- | --- |
| KCNQ1OT1 | Forward | TGGGGAAGGCTGCTTATTCG |
|  | Reverse | CGGAACCACTGTAGACCCAC |
| mmu-miR-421-3p | Forward | GGCGCATCAACAGACATT |
|  | Reverse | GTGCAGGTCCGAGGT |
| hsa-miR-421 | Forward | CTCACTCACATCAACAGACATTAATT |
|  | Reverse | TATGGTTTTGACGACTGTGTGAT |
| mTOR | Forward | GACCCCACTTCTCTTCGCAA |
|  | Reverse | CATAGTCAGGAGCCATCCGC |
| Col-1 | Forward | AGCTCGATACACAATGGCCT |
|  | Reverse | CCTATGACTTCTGCGTCTGG |
| RUNX2 | Forward | GGGACTGTGGTTACCGTCAT |
|  | Reverse | ATAACAGCGGAGGCATTTCG |
| ALP | Forward | GCACCTGCCTTACCAACTCT |
|  | Reverse | GTGGAGACGCCCATACCATC |
| OCN | Forward | TCTGACCTCACAGATGCCAAG |
|  | Reverse | AGGGTTAAGCTCACACTGCT |
| GAPDH | Forward | TGTCTCCTGCGACTTCAACA |
|  | Reverse | GGTGGTCCAGGGTTTCTTACT |
| U6 | Forward | CAGCACATATACTAAAATTGGAACG |
|  | Reverse | ACGAATTTGCGTGTCATCC |

*KCNQ1OT1, potassium voltage-gated channel subfamily Q member 1 overlapping transcript 1; mmu-miR-421-3p, mouse microRNA-421-3p; hsa-miR-421, human microRNA-421; mTOR, mammalian target of rapamycin; Col-1, collagen 1; RUNX2, runt-related transcription factor 2; ALP, alkaline phosphatase; OCN, osteocalcin; GAPDH, glyceraldehyde-3-phosphate dehydrogenase.
