# Supplementary material for: Long non-coding RNA KCNQ1OT1 alleviates postmenopausal osteoporosis by modulating miR-421-3p/mTOR axis
Source: Sci Rep. 2023 Feb 9;13:2333. doi: 10.1038/s41598-023-29546-4 (PMC9911397; doi:10.1038/s41598-023-29546-4)
Supplement: Supplementary file 3 — Supplementary Information 3. [file 41598_2023_29546_MOESM3_ESM.pdf]

NOTE: Please save this file locally before filling in the table, DO NOT work on the file within your internet browser as changes will not be saved. Adobe Acrobat Reader (available free here) is recommended for completion.

The ARRIVE guidelines 2.0: author checklist

The ARRIVE Essential 10

These items are the basic minimum to include in a manuscript. Without this information, readers and reviewers cannot assess the reliability of the findings.

Item		Recommendation	Section/line number, or reason for not reporting
**Study design**	1	For each experiment, provide brief details of study design including:	
		a. The groups being compared, including control groups. If no control group has been used, the rationale should be stated.	There are four groups of C57 mice including the control group. In section 'Treatment of Model Mice' of Page 18.
		b. The experimental unit (e.g. a single animal, litter, or cage of animals).	A single femur. In section 'Treatment of Model Mice' of Page 18.
**Sample size**	2	a. Specify the exact number of experimental units allocated to each group, and the total number in each experiment. Also indicate the total number of animals used.	In section 'Animal Ovariectomy (OVX)-Induced Osteoporosis Model' 'Treatment of Model Mice' of Page 16 and 18.
		b. Explain how the sample size was decided. Provide details of any *a priori* sample size calculation, if done.	According to the number of experimental animals in each group specified in most research papers, n=3.
**Inclusion and exclusion criteria**	3	a. Describe any criteria used for including and excluding animals (or experimental units) during the experiment, and data points during the analysis. Specify if these criteria were established *a priori*. If no criteria were set, state this explicitly.	N/A
		b. For each experimental group, report any animals, experimental units or data points not included in the analysis and explain why. If there were no exclusions, state so.	there were no excluded animals.
		c. For each analysis, report the exact value of *n* in each experimental group.	n=3. In section 'Animal Ovariectomy (OVX)- Induced Osteoporosis Model' 'Treatment of Model Mice' of Page 16 and 18.
**Randomisation**	4	a. State whether randomisation was used to allocate experimental units to control and treatment groups. If done, provide the method used to generate the randomisation sequence.	Yes. Number each mouse and group them by random number table.
		b. Describe the strategy used to minimise potential confounders such as the order of treatments and measurements, or animal/cage location. If confounders were not controlled, state this explicitly.	The experiment ruled out confounding factors
**Blinding**	5	Describe who was aware of the group allocation at the different stages of the experiment (during the allocation, the conduct of the experiment, the outcome assessment, and the data analysis).	Only the operators know the grouping.
**Outcome measures**	6	a. Clearly define all outcome measures assessed (e.g. cell death, molecular markers, or behavioural changes).	In section 'micro-CT Analysis' and 'histologic and Immunohistochemistry (IHC) Staining' of Page 18.
		b. For hypothesis-testing studies, specify the primary outcome measure, i.e. the outcome measure that was used to determine the sample size.	N/A
**Statistical methods**	7	a. Provide details of the statistical methods used for each analysis, including software used.	In section 'micro-CT Analysis', 'histologic and Immunohistochemistry (IHC) Staining' and 'statistical analysis' of Page 18 and 19.
		b. Describe any methods used to assess whether the data met the assumptions of the statistical approach, and what was done if the assumptions were not met.	In section 'statistical analysis' of Page 18 and 19.
**Experimental animals**	8	a. Provide species-appropriate details of the animals used, including species, strain and substrain, sex, age or developmental stage, and, if relevant, weight.	In section' Animal Ovariectomy (OVX)-Induced Osteoporosis Model' of Page 17.
		b. Provide further relevant information on the provenance of animals, health/immune status, genetic modification status, genotype, and any previous procedures.	In section' Animal Ovariectomy (OVX)-Induced Osteoporosis Model' of Page 17.
**Experimental procedures**	9	For each experimental group, including controls, describe the procedures in enough detail to allow others to replicate them, including:	All of them are showed in section 'Animal Ovariectomy (OVX)- Induced Osteoporosis Model' and 'Treatment of Model Mice' of Page 16 and 18.
		a. What was done, how it was done and what was used.	
		b. When and how often.	
		c. Where (including detail of any acclimatisation periods).	
		d. Why (provide rationale for procedures).	
**Results**	10	For each experiment conducted, including independent replications, report:	In Figure 8.
		a. Summary/descriptive statistics for each experimental group, with a measure of variability where applicable (e.g. mean and SD, or median and range).	In Figure 8.
		b. If applicable, the effect size with a confidence interval.	

The Recommended Set

These items complement the Essential 10 and add important context to the study. Reporting the items in both sets represents best practice.

Item		Recommendation	Section/line number, or reason for not reporting
**Abstract**	11	Provide an accurate summary of the research objectives, animal species, strain and sex, key methods, principal findings, and study conclusions.	Relative information are showed in section ' 'Animal Ovariectomy (OVX)- Induced Osteoporosis Model' and 'Treatment of Model Mice' of Page 16 and 18.
**Background**	12	a. Include sufficient scientific background to understand the rationale and context for the study, and explain the experimental approach.	Relative information are mentioned in section 'introduction' and 'discussion'.
		b. Explain how the animal species and model used address the scientific objectives and, where appropriate, the relevance to human biology.	In section' Animal Ovariectomy (OVX)-Induced Osteoporosis Model' of Page 17.
**Objectives**	13	Clearly describe the research question, research objectives and, where appropriate, specific hypotheses being tested.	Relative information are mentioned in section 'introduction' and 'abstract'.
**Ethical statement**	14	Provide the name of the ethical review committee or equivalent that has approved the use of animals in this study, and any relevant licence or protocol numbers (if applicable). If ethical approval was not sought or granted, provide a justification.	In section 'Ethics declarations' of Page 19.
**Housing and husbandry**	15	Provide details of housing and husbandry conditions, including any environmental enrichment.	Five mice in a cage, which raised in the Animal Experiment Center of Qilu Hospital of Shandong University
**Animal care and monitoring**	16	a. Describe any interventions or steps taken in the experimental protocols to reduce pain, suffering and distress.	All of them are mentioned in section 'Animal Ovariectomy (OVX)-Induced Osteoporosis Model' and 'Treatment of Model Mice' of Page 16 and 18.
		b. Report any expected or unexpected adverse events.	
		c. Describe the humane endpoints established for the study, the signs that were monitored and the frequency of monitoring. If the study did not have humane endpoints, state this.	
**Interpretation/ scientific implications**	17	a. Interpret the results, taking into account the study objectives and hypotheses, current theory and other relevant studies in the literature.	In section 'discussion'.
		b. Comment on the study limitations including potential sources of bias, limitations of the animal model, and imprecision associated with the results.	In section 'discussion'.
**Generalisability/ translation**	18	Comment on whether, and how, the findings of this study are likely to generalise to other species or experimental conditions, including any relevance to human biology (where appropriate).	In section 'discussion'.
**Protocol registration**	19	Provide a statement indicating whether a protocol (including the research question, key design features, and analysis plan) was prepared before the study, and if and where this protocol was registered.	In section 'Animal Ovariectomy (OVX)- Induced Osteoporosis Model' 'Treatment of Model Mice' of Page 16 and 18.
**Data access**	20	Provide a statement describing if and where study data are available.	In section 'Data Availability' of Page 19.
**Declaration of interests**	21	a. Declare any potential conflicts of interest, including financial and non-financial. If none exist, this should be stated.	In section 'Conflicts of Interest' of Page 19.
		b. List all funding sources (including grant identifier) and the role of the funder(s) in the design, analysis and reporting of the study.	In section 'Funding' of Page 20.
